# Selective Activation of CNS and Reference *PPARGC1A* Promoters Is Associated with Distinct Gene Programs Relevant for Neurodegenerative Diseases

**DOI:** 10.3390/ijms22073296

**Published:** 2021-03-24

**Authors:** Markus Kwik, Stefan Hainzl, Jan Oppelt, Boris Tichy, Ulrich Koller, Emanuele Bernardinelli, Markus Steiner, Greta Zara, Charity Nofziger, Serge Weis, Markus Paulmichl, Silvia Dossena, Wolfgang Patsch, Selma M. Soyal

**Affiliations:** 1 Institute of Pharmacology and Toxicology, Paracelsus Medical University, Strubergasse 18, 5020 Salzburg, Austria; markus.kwik@live.at (M.K.); e.bernardinelli@pmu.ac.at (E.B.); gzara@coh.org (G.Z.); silvia.dossena@pmu.ac.at (S.D.); 2 EB House Austria, Research Program for Molecular Therapy of Genodermatoses, Department of Dermatology and Allergology, University Hospital of the Paracelsus Medical University, 5020 Salzburg, Austria; s.hainzl@salk.at (S.H.); u.koller@salk.at (U.K.); 3 CEITEC-Central European Institute of Technology, Masaryk University, Kamenice 5, 62500 Brno, Czech Republic; jan.oppelt@mail.muni.cz (J.O.); boris.tichy@ceitek.muni.cz (B.T.); 4 Department of Pathology and Laboratory Medicine, Perlman School of Medicine, University of Pennsylvania, 614 Stellar-Chance Labs, 422 Curie Blvd, Philadelphia, PA 19104-6100, USA; 5 Third Medical Department, Cancer Research Institute, Paracelsus Medical University, Cancer Cluster Salzburg, 5020 Salzburg, Austria; mark.steiner@salk.at; 6 PharmGenetix GmbH, 5081 Niederalm, Austria; charity.nofziger@pharmgenetix.com; 7 Division of Neuropathology, Neuromed Campus, Kepler University Hospital, 4020 Linz, Austria; serge.weis@kepleruniklinikum.at; 8 Department of Personalized Medicine, Humanomed, 9020 Klagenfurt, Austria; paulmichl@me.com

**Keywords:** *PPARGC1A*, PGC-1α, CNS-specific transcripts and isoforms, CRISPR, RNA sequencing, RNA expression, exon usage, neurodegenerative diseases

## Abstract

The transcriptional regulator peroxisome proliferator activated receptor gamma coactivator 1A (PGC-1α), encoded by *PPARGC1A*, has been linked to neurodegenerative diseases. Recently discovered CNS-specific *PPARGC1A* transcripts are initiated far upstream of the reference promoter, spliced to exon 2 of the reference gene, and are more abundant than reference gene transcripts in post-mortem human brain samples. The proteins translated from the CNS and reference transcripts differ only at their N-terminal regions. To dissect functional differences between CNS-specific isoforms and reference proteins, we used clustered regularly interspaced short palindromic repeats transcriptional activation (CRISPRa) for selective endogenous activation of the CNS or the reference promoters in SH-SY5Y cells. Expression and/or exon usage of the targets was ascertained by RNA sequencing. Compared to controls, more differentially expressed genes were observed after activation of the CNS than the reference gene promoter, while the magnitude of alternative exon usage was comparable between activation of the two promoters. Promoter-selective associations were observed with canonical signaling pathways, mitochondrial and nervous system functions and neurological diseases. The distinct N-terminal as well as the shared downstream regions of PGC-1α isoforms affect the exon usage of numerous genes. Furthermore, associations of risk genes of amyotrophic lateral sclerosis and Parkinson’s disease were noted with differentially expressed genes resulting from the activation of the CNS and reference gene promoter, respectively. Thus, CNS-specific isoforms markedly amplify the biological functions of *PPARGC1A* and CNS-specific isoforms and reference proteins have common, complementary and selective functions relevant for neurodegenerative diseases.

## 1. Introduction

Alternative splicing and/or promoter usage occur in the great majority of mammalian genes and are key mechanisms in transcriptional regulation and generation of protein diversity [1,2,3]. Peroxisome proliferator activated receptor gamma coactivator 1A (PGC-1α), encoded by *PPARGC1A,* is a versatile transcriptional coactivator that participates in the regulation of many transcriptional programs [4,5,6]. In addition to co-activating numerous transcription factors, PGC-1α is also involved in alternative splicing [7]. Moreover, several PGC-1α isoforms that result from alternative splicing have been characterized. N-terminal truncated (NT)-PGC-1α is one such isoform that displays functional differences in comparison to the full-length protein [8,9,10]. Tissue-selective PGC-1α isoforms resulting from alternative promoter usage have been identified as well. Two novel promoters in the skeletal muscle that are differentially regulated in response to fasting and exercise are located 14 kbp upstream of the reference promoter [11,12]. In the human liver, transcription from an alternative promoter located in intron 2 of the reference gene (RG) [13] generates a 75 kDa protein that shows subtle differences in co-activation selectivity compared to the reference protein [14]. We discovered CNS-specific *PPARGC1A* mRNAs that are transcribed from a novel promoter located ~583 kbp upstream of the reference promoter. These transcripts are more abundant than RG mRNAs in human post-mortem brain samples and are partially conserved in rodents [10].

Functional studies in animal or cell culture models implicate PGC-1α in clinically distinct neurodegenerative diseases including Huntington’s disease (HD), Parkinson’s disease (PD), Alzheimer’s disease (AD), and amyotrophic lateral sclerosis (ALS) [15,16,17,18,19]. Immune-mediated CNS disorders such as multiple sclerosis have been linked to PGC-1α as well [20,21]. RNA sequencing and genetic studies in humans also suggested that the *PPARGC1A* locus modulates the risk of AD, HD, PD, and ALS [10,22,23,24,25,26,27,28]. As some of these associations included the CNS-specific region of the *PPARGC1A* locus, CNS-specific isoforms and/or their control may be involved in these disorders.

We recently compared the regulation of the CNS and RG *PPARGC1A* promoters in human neuronal cell lines and identified similarities, but also substantial differences in the signaling pathways that converge at the two promoters [29]. To characterize the function of CNS-specific isoforms, we studied qualitative and quantitative differences in mRNA expression that result from selective endogenous activation of the CNS or RG promoters in SH-SY5Y cells. We used an application of the versatile gene-editing system clustered regularly interspaced short palindromic repeats (CRISPR) and CRISPR-associated proteins (Cas) [30], which activates transcription and has been termed CRISPRa [31,32]. The method relies on a Cas9 variant termed dCas9. The variant enzyme harbors two amino acid substitutions (D10A and H840A) causing inactivation of endonucleolytic activities, but its capacity to bind to the target DNA based on the complementarity of sgRNA sequences is retained. dCas9 has been functionalized by fusion with transcriptional activator domains that induce the expression of genes by sgRNAs targeting sequences near their transcription start sites [33]. Noteworthy, the induction of endogenous genes via promoters in their native context provides insight into naturally occurring splice variants and cis- and trans-regulatory effects on transcription and translation. We report here that the selective activation of the CNS and RG promoters and hence their resulting repertoire of PGC-1α isoforms generates overlapping as well as substantially divergent gene programs in SH-SY5Y cells.

## 2. Results

### 2.1. Endogenous Activation of RG and CNS PPARGC1A Promoters

We used an effective transcriptional activation system consisting of dCas9 fused to a potent tripartite activator called VPR encoding herpes simplex virus protein 16 (VP64), the activation domain of the p65 subunit of nuclear factor NF-κB and the replication and transcription activator (RTA) of the γ-herpesvirus family [33]. To verify lentiviral transduction of SH-SY5Y cells, dCas9-VPR encoding transcripts were amplified by qRT-PCR from total RNA. Sequencing of amplicons revealed the dCas9-VPR fragment, as expected (data not shown). Clonal SH-SY5Y cell lines expressing dCas9-VPR were obtained by puromycin selection followed by single cell sorting and culture. Out of 96 wells, we observed cell growth in 25 wells. Three clones termed SH-SY5Y-dCas9-VPR-v1 to –v3 were further characterized. Clonal lines v1-v3 showed similar levels of dCas9-VPR transcripts (Figure S1).

**Figure 1:**
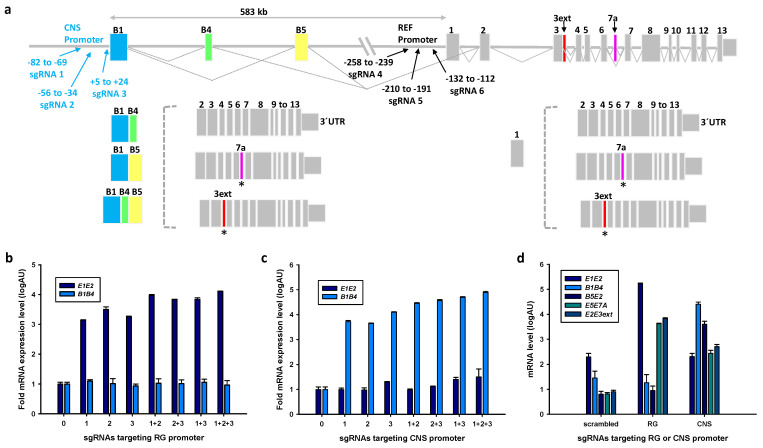
The CNS and reference gene (RG) promoters are selectively activated by specific single guide RNAs (sgRNAs) designed to target them. (**a**) Schematic representation of the *PPARGC1A* locus showing the CNS and RG promoters (top), locations of sgRNAs used for transfections; CNS-specific exons *B1*, *B4* and *B5* in color; the structure of the RG is displayed on the right; CNS-specific transcripts and RG transcripts are shown below; * pink or red lines refer to alternatively spliced transcripts encoding stop codons in exon 7A or in an extension of exon 3, respectively. (**b**,**c**) Selective effects of individual sgRNAs targeting the RG or CNS promoters on transcription initiation; individual sgRNAs or their mixtures were transfected into clonal SH-SY5Y cells expressing CRISPR-associated deactivated (dCas9) protein fused to the tripartite transcriptional activator VPR and levels of *E1E2* and *B1B4* transcripts selective for RG or CNS promoter activation were measured by qRT-PCR 36 h after transfection. Log-fold levels are expressed relative to transcript levels of the clonal cells transfected with scrambled sgRNA. Interactions of all sgRNA mixtures shown were significant (*p* < 0.001) when the use of three, two, or one sgRNA was compared. (**d**) Effects of sgRNA transfections on CNS-specific and RG transcript levels and levels of transcripts encoding truncated isoforms and initiated at either promoter.

We next focused on the selectivity of CNS and RG promoter activation by sgRNAs. The distance between the two promoters is 583 kbp, which approximates 6-fold the size of the RG. Transcripts generated from the two promoters differ in that CNS-specific transcripts contain exons B1, B4 and/or B5, which are spliced to exon 2 of the RG (Figure 1a, Figure S2). Locations of sgRNAs used, their sequences, specificity scores and potential homologies with 0 to 3 sequence mismatches are shown in Figure 1a, Tables S1 and S2a,b. We scrutinized the specificities of sgRNAs by comparing expression levels of off-target genes after transfections with scrambled and CNS or RG directed sgRNAs (Table S2a,b). Among the potential off-targets with two mismatches of RG sgRNAs, an upregulation of *LDLR* relative to scrambled sgRNA was noted (log2-fold increase 0.219, adjusted *p* = 0.014), but a similar upregulation was observed with CNS promoter directed sgRNAs. For all other mismatches of RG sgRNAs, the expression changes were associated with comparable changes induced by sgRNAs targeting the CNS promoter. Similarly, among the potential off-targets of CNS promoter directed sgRNAs, altered expression was also noted after activation of the RG promoter with the exception of *FGFR3* mRNA which showed a nominal decrease after activation with CNS sgRNAs (log2-fold change 0.0423, adjusted *p* = 0.01). Thus, the up- or down-regulation of the respective genes by activation of either promoter makes off-target effects unlikely.

Transfections of sgRNAs were optimized for plasmid DNA amount and volume of transfection reagent. Time course experiments showed the highest expression levels of CNS-specific and RG transcripts 38 h after transfection with the respective sgRNA mixtures (data not shown). To determine transfection efficiency, the six plasmids each co-expressing one sgRNA targeting the RG or the CNS promoters and EGFP were used. Transfection efficiencies of SH-SY5Y-dCas9-VPR-v1 or –v2 cells were similar for plasmids encoding scrambled, RG or CNS-specific sgRNAs as judged by the number of cells expressing EGFP and ranged between 18 and 25% (Figure S3).

Individual plasmids encoding sgRNAs or mixtures thereof were transfected into SH-SY5Y-dCas9-VPR-v1 and the expression levels of CNS-specific *B1B4* and RG-specific *E1E2* transcripts were determined. These studies indicated high specificity of the selected sgRNAs for their respective promoter. Synergistic effects were noted for mixtures of two or three plasmids encoding sgRNAs (all *p*-values < 0.001) targeting their corresponding promoters (Figure 1b,c). We next quantified the *PPARGC1A* transcripts encoding the isoforms shown in Figure 1a after activation of RG or CNS promoters. In SH-SY5Y cells the RG transcript levels were higher than CNS transcript levels (Figure 1d). We aimed to activate the RG and CNS promoters to a similar extent (800 to 1000-fold) as judged from the increase in levels of cells transfected with scrambled sgRNAs. Transfections with a mix of plasmids expressing all three sgRNAs targeting the RG promoter substantially increased the RG transcript *E1E2* and transcripts encoding the two splicing variants NT-PGC-1α and E3extended, but failed to induce *B1B4* and *B5E2* transcripts. To activate the CNS promoter ~1000-fold, only two plasmids encoding distinct sgRNAs were required. These transfections resulted in increases of the CNS-specific transcripts *B1B4* and *B5E2*, and smaller increases of transcripts encoding the two splicing variants NT-PGC-1α and E3extended, while the level of the RG transcript *E1E2* was not increased (Figure 1d). Thus, transcripts encoding the two splicing variants are generated from both promoters.

**Figure 2:**
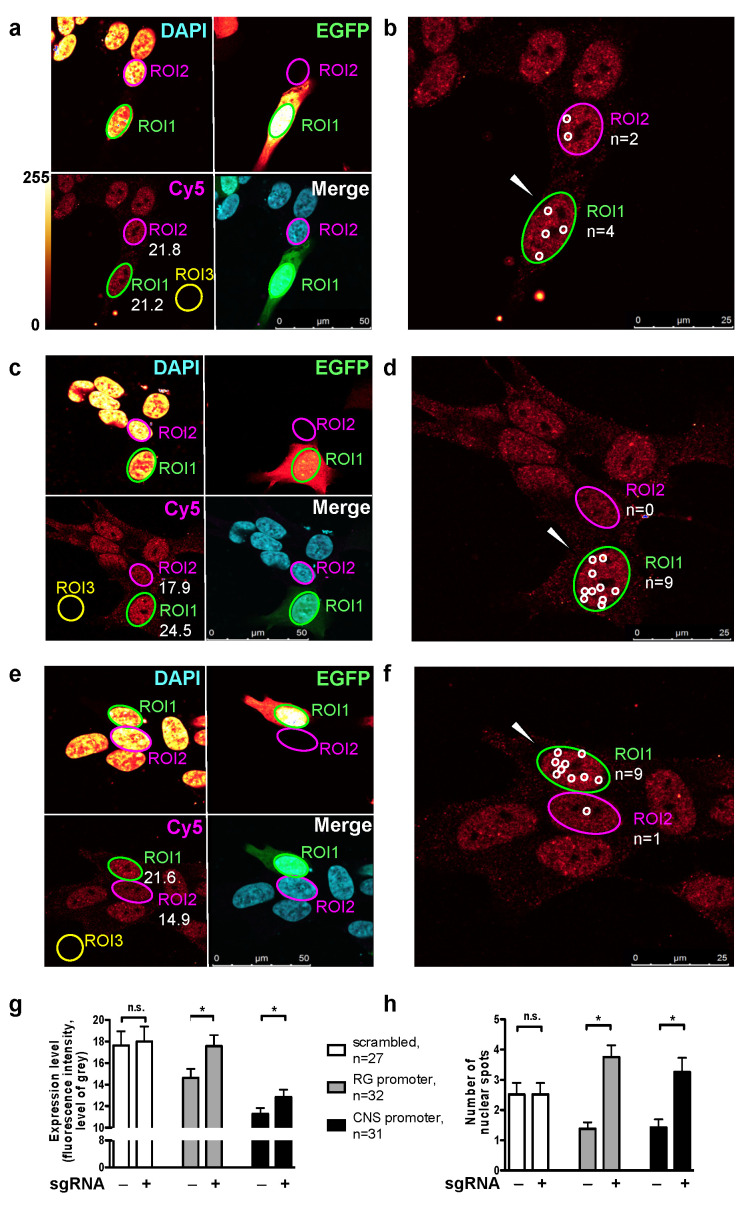
Targeted activation of the RG or CNS promoters increases the expression of PGC-1α proteins in sgRNA transfected clonal SH-SY5Y cells expressing dCas9-VPR. (**a**,**c**,**e**) From top left to bottom right: fluorescent signal of cellular nuclei counterstained with 4′,6-diamidino-2-phenylindole (DAPI), EGFP, Cy5 (antibody directed against the N-terminal region of reference/CNS PGC-1α) and the corresponding merged image in cells transfected with scrambled sgRNA or sgRNAs targeting the RG and CNS *PPARGC1A* promoters, respectively. Numbers in the lower left section of panels refer to the fluorescence intensity of Cy5 (levels of gray) determined in regions of interest (ROIs) corresponding to the nucleus of adjacent transfected (EGFP-expressing, ROI1) and mock-transfected (not expressing EGFP, ROI2) cells. ROIs depicted outside the cells (ROI3) served to determine the background fluorescence. The fluorescence intensity scale shown in (**a**) also applies to (**c**,**e**). (**b**,**d**,**f**) Magnification of the lower left section of panels (**a**,**c**,**e**); the nuclear spots with a fluorescence intensity >30 levels of gray in adjacent transfected (white arrowhead) and mock-transfected cells are circled. (**g**) Mean fluorescence intensity and (**h**) number of nuclear spots per nucleus in cells transfected with scrambled sgRNAs, RG- and CNS specific sgRNAs and mock-transfected cells. Means (SE),* *p* < 0.001, two-tailed, paired Student’s *t* test; n corresponds to the number of adjacent transfected and mock-transfected cell pairs; n.s., not significant.

Immunocytochemistry was used to estimate the levels of PGC-1α proteins. To determine the selectivity of antibodies, SH-SY5Y-dCas9-VPR positive clonal cells were transfected with plasmids encoding E1-7A-EGFP, B4-7A-EGFP, and B5-13-EGFP fusion proteins. The first two plasmids encode the NT-forms of the RG and CNS-specific B4 protein, while the last plasmid encodes the full-length CNS-specific B5 protein [10]. Among several antibodies tested, a commercial mouse monoclonal antibody (#ST1202, Calbiochem) against the N-terminal region (amino acids 1–120) of reference PGC-1α was selected, which showed positive immunochemical reactions for both the reference and CNS-specific isoforms which contain the amino acids of the reference protein encoded by exon 2 (starting at amino acid residue 19) (Figure S4). SH-SY5Y-dCas-VPR cells were transfected with plasmids co-expressing EGFP and scrambled sgRNAs or sgRNAs targeting the RG or CNS promoters for 40 h. Cy5 fluorescence intensities and average levels of gray in regions of interest (ROIs) harboring EGFP expressing transfected cells and adjacent mock-transfected cells were compared. Examples of confocal scans for transfections with scrambled and CNS or RG promoter targeting sgRNAs are shown in Figure 2a,c,e. Transfections with sgRNAs targeting the RG or CNS promoters resulted in a significantly higher level of fluorescence intensities in the nuclei in comparison to mock-transfections (Figure 2g), while no differences were observed between cells mock-transfected or transfected with scrambled sgRNAs. We also counted the number of spots with a fluorescence intensity > 30 levels of gray in these cells (Figure 2b,d,f). Such spots are likely nuclear speckles known to be involved in various nuclear functions including splicing [34]. Again, no effect was observed for scrambled sgRNA, but higher counts were obtained for cells transfected with sgRNAs targeting the RG or CNS promoters than in the respective mock-transfected cells (Figure 2h). Thus, activated RG and CNS promoters induced the expressions of the respective PGC-1α isoforms. However, the increase at the protein level was likely much lower than the increase at the transcript level, even though the methods used do not allow absolute quantification. Numerous mechanisms may account for this difference [35].

To evaluate the metabolic activity of cells, we used the MTT assay. After 24 and 40 h of transfection, cells transfected with CNS- and RG-specific sgRNAs showed higher activities in the MTT assay compared with cells transfected with scrambled sgRNA (Figure S5). Hence, toxic effects resulting from CNS or RG promoter activation were unlikely.

### 2.2. RNA Sequencing

#### 2.2.1. Effects of Selective Promoter Activation on RNA Expression Levels

**Figure 3:**
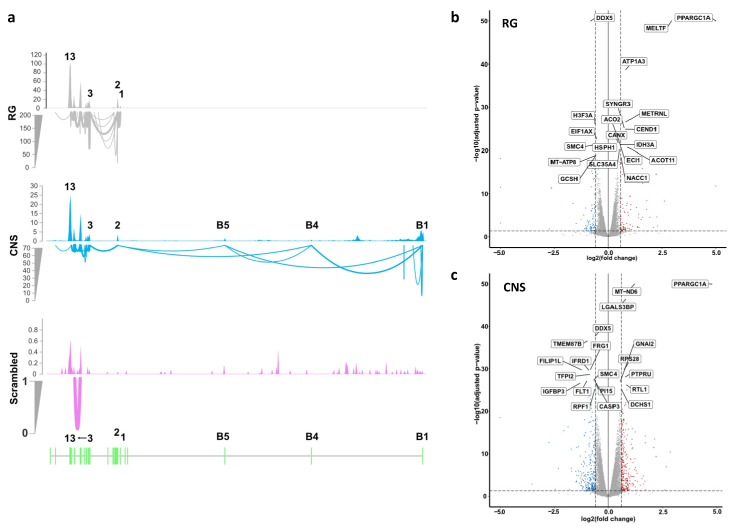
RNA sequencing confirms the predicted structure of transcripts generated by activation of the RG or CNS promoters and reveals differentially expressed genes (DEGs) for either promoter activation. (**a**) Sashimi plots of *PPARGC1A* transcripts generated by transfection of clonal SH-SY5Y cells expressing dCas9-VPR with sgRNAs activating the RG or the CNS promoters or with scrambled sgRNAs, each merged from three biological replicates; read densities across exons are normalized to obtain comparable measures of expression above the *x*-axis and normalized single-end junction reads are shown as arcs below the *x*-axis; structure of visualized exons is shown at the bottom. (**b**,**c**) Volcano plots of DEGs in cells with RG or CNS activated promoters, respectively, in comparison to cells transfected with scrambled sgRNAs. The top 20 most DEGS are highlighted.

Exploratory analysis by multidimensional scaling (MDS) revealed separation of the leading biological coefficient of variation (BCV) for the activation of the CNS and the RG promoters and controls transfected with scrambled sgRNAs (Figure S6). Furthermore, as already established previously by PCR analyses, the sequencing data showed that activation of the RG promoter induced transcripts starting at exon 1, while activation of the CNS promoter induced transcripts initiated at exon *B1* and spliced to *B4* and/or *B5* and exon 2, but lacking exon 1. Thus, the transcripts generated included *B1B4B5E2*-, *B1B5E2*-, and *B1B4B5E2*-(Figure 3a, Figures S7 and S8). Levels of *PPARGC1A* RG transcripts were ~4 times higher than *PPARGC1A* CNS-specific transcripts. This finding is consistent with the lower basal CNS-specific transcript levels in SH-SY5Y and a similar fold-activation of the two promoters. The top 20 most differentially expressed genes (DEGs) after RG or CNS promoter activation are highlighted in Volcano (Figure 3b,c) or MA plots (Figure S9).

**Figure 4:**
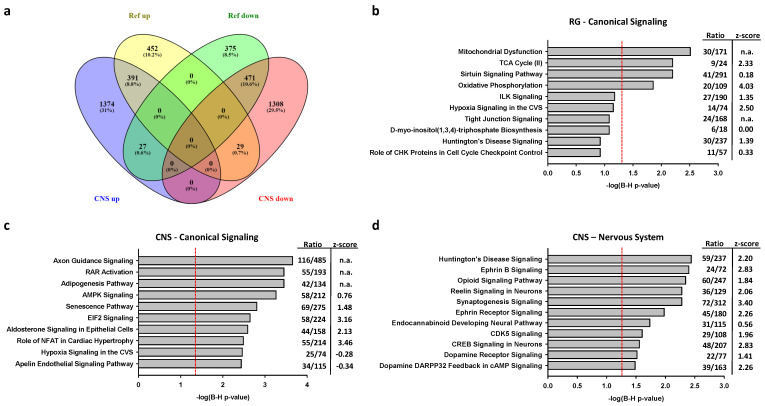
Differentially expressed genes resulting from activation of the RG or CNS promoters reveal promoter selectivity with partial overlap. (**a**) Venn diagram of differentially expressed genes (DEGS) produced by transfection of clonal SH-SY5Y cells expressing dCas9-VPR with sgRNAs activating the RG or the CNS promoters are compared with cells transfected with scrambled sgRNAs. (**b**,**c**) Top canonical signaling pathways for DEGs after RG or CNS promoter activation, respectively. (**d**) Top nervous system signaling pathways for DEGs after CNS promoter activation; red lines refer to adjusted *p*-values of 0.05; *z*-scores > 2.0 or <−2.0 are significant and indicate the direction for the expected entity; n.a, not available; B-H, Bernini-Hochberg adjusted *p*-values for multiple testing.

In comparison to SH-SY5Y-dCas-VPR cells transfected with scrambled sgRNAs, cells transfected with CNS or RG directed sgRNAs exhibited differential expression of 6409 or 4281 genes, respectively, at an adjusted *p*-level <0.05. Further restriction of cutoffs to 0.263 log2-fold changes in expression levels relative to the expression levels after transfections with scrambled sgRNAs resulted in altered expression levels of 1745 genes (872 up- and 873 down-regulated) and 3600 genes (1792 up- and 1808 down-regulated) after activation of the RG and the CNS promoters, respectively (Figure 4a). From the 1792 genes upregulated by CNS promoter activation, 391 also were upregulated by RG promoter activation. From the 1808 genes downregulated by CNS promoter activation, 471 were also downregulated by activation of the RG promoter. Thus, 862 genes were co-regulated, while 56 genes were contra-regulated, i.e., upregulated by activation of one, but repressed by activation of the other promoter. Expression changes of several genes were verified by PCR (Figure S10).

#### 2.2.2. Associations of Selective Promoter Activation on Pathways

The top 10 canonical ingenuity signaling pathways associated with DEGs resulting from RG or CNS promoter activation are shown in Figure 4b,c. The four RG promoter associated pathways that maintained a level of *p* < 0.05 after adjustment for multiple comparison included mitochondrial dysfunction, TCA cycle II, the sirtuin signaling pathway and oxidative phosphorylation. Unexpectedly, these pathways revealed no or only weak associations with CNS promoter activated genes. Importantly, the latter genes included axon guidance signaling (Figure 4c). To further characterize the differential effects of RG and CNS promoter activation, the genes that were co- or contra-regulated by the CNS and RG promoters were excluded. Enrichment analyses of the reduced gene sets revealed that the top five pathways did not change for either promoter activation, thereby reinforcing the concept of target selectivity for the reference and CNS-specific PGC-1α proteins. The genes differentially expressed by CNS promoter activation revealed significant associations with pathways relevant for neurobiology and nervous system signaling (Figure 4d), while no such associations were observed with RG promoter activation after adjustment for multiple testing.

**Figure 5:**
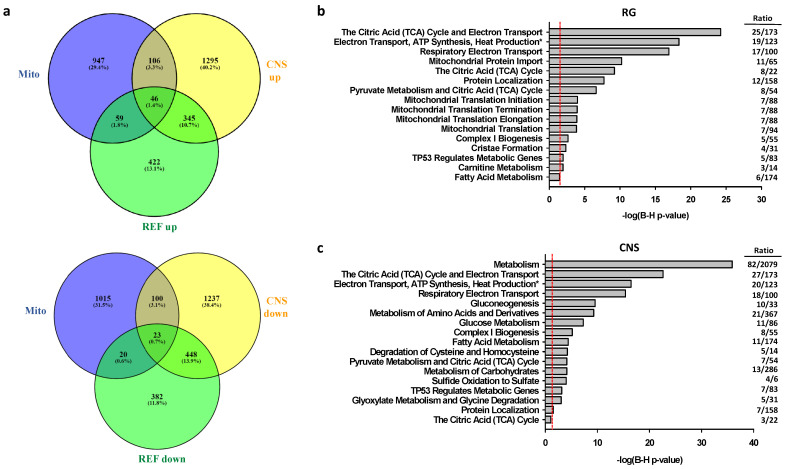
RG and CNS promoter activation induces similar and different changes in mitochondrial genes. (**a**) Venn diagrams of mitochondrial up- (top) or down-regulated genes (bottom); (**b**,**c**) Reactome pathways deduced from enrichment analyses of up-regulated mitochondrial genes after activation of RG or CNS promoters; * full name: Respiratory electron transport, ATP synthesis by chemiosmotic coupling, and heat production by uncoupling proteins.

As the absence of associations between CNS promoter activated DEGs and classical mitochondrial pathways was unexpected, we interrogated the activation/repression of mitochondrial genes (mito-genes) in greater detail (Figure 5a). Mito-genes were enriched in both the CNS and RG promoter activated gene sets (*p* < 0.00001), but the proportion of mito-genes was higher in the RG than in the CNS promoter activated genes (12 vs. 8.5%, *p* = 0.0103, two-tailed). Activation of RG or CNS promoters tended to reduce the proportion of mito-genes in the repressed gene sets (4.9% vs. 6.8%, *p* = 0.0865). Among CNS promoter activated genes, expression levels of 11 genes encoded by mitochondria (MT-genes) of complex I, II, and IV increased, while levels of *MT-ATP8* decreased. Among RG promoter activated genes, *MT-ND4L*, *MT-ND5,* and *MT-N6* of complex I increased, while *MT-ATP6* and *MT-ATP8* of complex V were repressed (Table S3). Enrichment analyses of the 152 CNS and the 105 RG upregulated mito-genes showed associations of both gene sets with the reactome pathway of respiratory electron transport, ATP synthesis by chemiosmotic coupling and heat production by uncoupling proteins. Interestingly, the association with metabolism was much stronger in CNS than in RG promoter activated mito-genes, while the opposite was true for the citric acid cycle. Significant associations with mitochondrial import, mitochondrial translation terms, cristae formation, and carnitine metabolism were only noted in RG promoter activated mito-genes, while associations with gluconeogenesis, glucose metabolism, metabolism of amino acids and derivatives and related pathways were only found in CNS promoter activated genes (Figure 5b,c).

**Figure 6:**
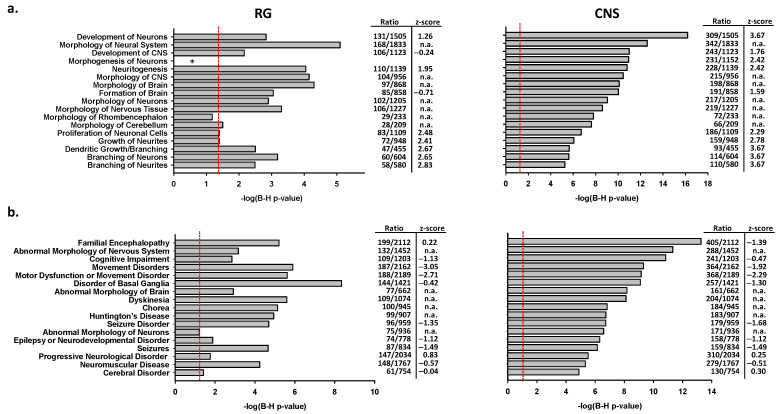
Targets of CNS-specific and reference PGC-1α proteins reveal comparable and distinct associations with neuronal function and neurological diseases. Top pathways in the ingenuity function ontology category “nervous system development and function” (**a**) or the disease category “neurological diseases” (**b**) for DEGs after RG or CNS promoter activation; the red line shows an adjusted *p*-value of 0.05; n.a., not available; * not among the top 40 associations listed by the program.

CNS-*PPARGC1A* transcripts in humans are expressed nearly exclusively in the CNS [10]. We therefore analyzed associations of gene sets in the ingenuity function ontology category “Nervous System Development and Function”. The number of genes included in this category divided by the number of DEGs was higher for the CNS than the RG promoter activated gene sets (0.210 vs. 0.1816, *p* = 0.0218, two-tailed). Removal of the overlapping DEGs showed similar results (0.2158 vs. 0.1721, *p* = 0.0102). Furthermore, the top associations of CNS and RG activated genes with functional terms ranked by their adjusted *p*-value did not correlate (*p* = 0.230, Spearman test). Indeed, the relative strengths of CNS and RG promoter activated gene associations showed major differences for the development of CNS and neurons as well as for the morphogenesis of neurons. Moreover, high z-scores (>2.0) were observed for the development of neurons in CNS promoter activated genes, while z-scores for terms comprising proliferation, growth, and branching of neurons, neurites, and dendrites were high in both CNS and RG promoter activated gene sets suggesting the expected activating effects within the respective entity (Figure 6a).

Enrichment analyses of CNS and RG promoter activated gene sets with entries included in the ingenuity ontology subset “Neurological Diseases” revealed associations with several broad terms such as cognitive impairment or movement disorders that include a number of diseases as well as narrow terms such as Huntington’s Disease (Figure 6b). The negative z-scores for movement disorders in both gene sets suggest an inhibitory effect on the disease phenotypes. Associations of disease terms and CNS or RG promoter activated gene sets ranked by *p*-values showed only a weak correlation (*p* = 0.0231, Spearman test).

To gain insight into physical interactions of proteins encoded by the gene sets defined in Figure 4a, we used the STRING database [36]. All but one gene set predicted more protein–protein interactions among themselves than random similar sized sets drawn from the genome. Thus, the proteins within our gene sets would be expected to be partially biologically connected. In addition, protein interactions of PGC-1α with other proteins also appeared to differ among the gene sets, but this observation needs to be cautiously interpreted, as STRING does not distinguish between the reference and other PGC-1α isoforms. Moreover, predicted local network clusters (STRING) showed no overlap between the gene sets that resulted from activation of the RG and CNS promoters (Table S4a–c). Finally, overall striking differences in network appearance were noted between the gene sets predicted for RG and CNS promoter activation (Figures S11–S14). One may therefore speculate that altered exon usage of *PPARGC1A* has a major impact on predicted protein networks.

#### 2.2.3. Associations of CNS or RG Promoter Activation with Neurodegenerative Disease Genes

Prompted by studies suggesting a role of reference- and/or CNS-PGC-1α in neurodegenerative diseases, we determined whether activation of the RG or CNS promoters modulated the expression of genes implicated in the genetic landscape of these diseases. A poly-glutamine tract of variable length that is encoded by CAG repeats in the *HTT* gene is the main cause of HD [37] and its length accounts for 70% of the variability in disease onset. Activation of the RG promoter resulted in upregulation of *HTT* (log2-fold change 0.375, adjusted *p* = 5.99 × 10^−7^), while the effect of CNS promoter activation did not reach significance (log2-fold change 0.115, adjusted *p* = 0.1415).

For ALS, AD, and PD, we analyzed effects of CNS and RG promoter activation on genes that were inferred to harbor risk loci identified by genome wide association studies (GWAS) or other converging evidence. DEGs with adjusted *p*-values <0.05 and cutoffs of 0.070 log2-fold changes were used. From 58 ALS risk genes [38,39,40,41,42], 12 were not or were only minimally expressed in SH-SY5Y cells [43]. Upon activation of the CNS promoter, 36 risk genes were differentially expressed (*p* = 0.0013, Fisher Exact Test, two-tailed). The respective gene count was 20 for activation of the RG promoter (*p* = 0.0171). For AD, 24 of 73 risk genes [44,45,46] were not expressed in SH-SY5Y cells. The analysis for CNS promoter activation was nominally significant (*p* = 0.0311). From 141 PD risk genes [47,48,49] 26 were not expressed in SH-SY5Y cells and 55 (*p* = 0.0456) or 48 (*p* < 0.0001) risk genes were differentially expressed by activation of the CNS or RG promoter (Table 1). Thus, the associations of CNS and RG promoter activated/repressed genes with ALS and PD, respectively, remained intact after the Bonferroni correction. Gene lists and log2-fold expression levels are shown in Tables S5–S7.

#### 2.2.4. Effects of Selective Promoter Activation on Exon-Usage

**Figure 7:**
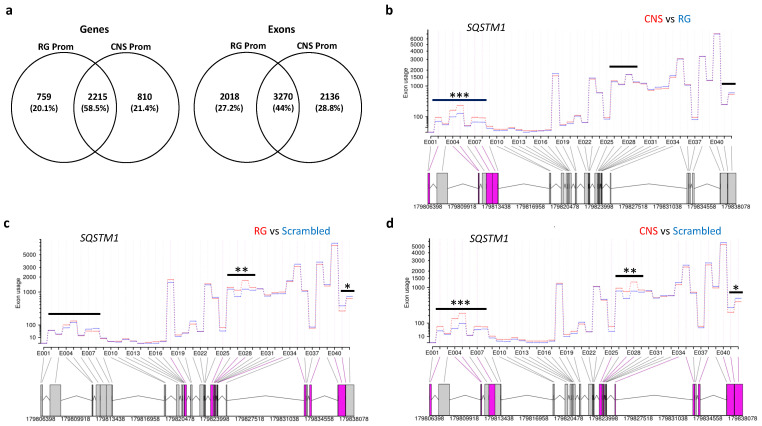
CNS-specific and reference PGC-1α proteins affect exon usage via distinct and similar mechanisms. (**a**) Venn diagram showing the number of genes (left) or exons (right) alternatively spliced after activation of the RG or the CNS promoters in comparison to cells transfected with scrambled sgRNAs. Exon usage of *SQSTM1* after RG and CNS promoter activation reveals similar changes to transfections of scrambled sgRNAs (control) and additional differences between each other; comparison between (**b**) CNS and RG promoter activation, (**c**) RG promoter activation and control and (**d**) CNS promoter activation and control; different exon usage between pairwise comparisons is indicated by purple boxes representing the affected exon bins; significant differences (*p* < 0.0001) are highlighted by stars; * and **, regions different between RG or CNS vs. scrambled; ***, region, different between CNS vs. RG or scrambled. The black lines refer to differences in any comparison.

As PGC-1α isoforms have been reported to selectively regulate multiple splicing events in the skeletal muscle [50], we studied the possible effects of CNS and RG promoter activation on differential exon usage relative to cells transfected with scrambled sgRNA. To enhance stringency, we used adjusted *p*-levels < 0.0001 and log2-fold changes of 0.5 for any exon as suggested [51]. The activation of RG or CNS promoters selectively affected exon usage of 2018 exons in 759 genes or 2136 exons in 811 genes, respectively, and alternative splicing was shared by activation of the two promoters in 3270 exons of 2215 genes (Figure 7a). Comparison of shared exons and genes indicated that 39 of the 2215 shared genes harbored one or more promoter selective changes in addition to the exon usage changes common for both promoters. For example, *SQSTM1,* which has been linked to ALS [52,53] showed very similar changes in the downstream exon bins for the comparisons between RG or CNS promoter activation with transfections of scrambled sgRNA. However, upstream exon bins were relatively increased in CNS promoter activation in comparison to controls (suggesting stimulation of an upstream promoter), while RG promoter activation and controls showed similar readouts in this region (Figure 7b,c,d). Examples of similar and distinct effects of CNS and RG promoter activation on exon usage are shown in Figures S15 and S16.

## 3. Discussion

Previously, we observed substantial differences in the regulation of the CNS and the RG promoters [29] and show now that the transcriptomes generated by selective activation of these promoters in a human cell model reveal marked differences. Importantly, CNS-specific isoforms most likely broaden the signaling response and protein diversity of *PPARGC1A* in the CNS. Our studies provide novel gene expression data that are relevant for neuronal functions and the pathogenesis of neurodegenerative diseases. Hence, the dataset will help to generate novel and testable hypotheses of biological and medical relevance.

As the CNS-specific transcripts are only partially conserved in rodents [10], we used undifferentiated SH-SY5Y cells that express, albeit at low levels, the transcripts encoding full-length CNS-specific isoforms [29]. The suitability of undifferentiated SH-SY5Y cells for experimental studies in neurodegenerative diseases is strongly supported by a recent disease-specific network approach [43]. To ascertain differences in gene programs, we used endogenous promoter activation by CRISPRa rather than ectopic expression of individual proteins. The former approach is expected to better reflect the in vivo situation and induces up-regulation of all promoter-specific transcripts (both known and perhaps yet unknown) concurrently. Even though aberrant transcription is generally not considered to be a large problem of the CRISPRa procedure [32,33], our comprehensive analysis made off-target effects unlikely. Endogenous activation of the CNS and RG promoters is expected to produce transcripts encoding full-length proteins and truncated isoforms. Proteins generated from the two promoters have distinct N-terminal regions, but are identical downstream of exon 2. Hence, the differences between the gene activation and repression programs can be attributed to their N-terminal domains, but the respective truncated isoforms may contribute to overall transcriptome differences. Our results are consistent with other studies showing distinct functions of PGC-1α isoforms [8,9,54].

In comparison to RG promoter activation, CNS promoter activation resulted in a greater number of DEGs, even though the level of CNS-specific *PPARGC1A* transcripts attained was lower than the level of *PPARGC1A* transcripts initiated at the RG promoter. An upstream open reading frame (uORF) located in the 5′ untranslated region of RG transcripts that represses translation may have contributed to the larger gene spectrum resulting from CNS promoter activation [55]. An uORF is also present in *B1B4B5* transcripts encoding the isoform B5-PGC-1α, but not in *B1B5* transcripts encoding the same protein or *B1B4* transcripts encoding the more abundant B4-PGC-1α isoforms (Soyal S, Patsch W, in preparation). While some overlap among the CNS and RG promoter activated or repressed genes was observed, most genes differed. This conclusion is highlighted by the distinct associations with canonical signaling pathways, mitochondrial pathways and physiological functions. Interestingly, transcript expression levels of MT-genes such as *MT-N2*, *MT-N3,* and *MT-CO3* appeared to differ upon activation of CNS and RG promoters. However, cautious interpretation of these data is necessary, as the contribution to mitochondrial translation appeared to differ as well.

The gene programs induced by CNS and RG promoter activation also include alternative splicing. Aberrant splicing has been linked to several neurodegenerative disorders [56,57]. As a large portion of splicing changes appeared to be shared by CNS and RG proteins, common structural features and/or effects of common downstream genes may be invoked. The C-terminal region of full-length PGC-1α isoforms harbors motifs characteristic of splicing factors including two arginine/serine-rich (RS) domains and an RNA recognition motif (RRM) [58]. RS domains interact with components of the spliceosome [59], and the RRM motif is present in many proteins involved in almost all aspects of RNA processing [60]. Deletion of these domains alters the splicing activity of PGC-1α on endogenous and synthetic minigenes [7,61]. However, another large portion of splicing events was selectively associated with the activation of the RG or CNS promoters. Hence, the underlying mechanism must be related to the discriminatory N-terminal sequences of reference and CNS-specific proteins. Current concepts suggest that pre-mRNA processing occurs co- rather than post-transcriptionally [62,63,64] and differential recruitment of nuclear co-activators likely plays a role in the selection of alternative splice sites of target genes [65]. The mechanisms involved may include distinct interactions of the CNS-specific and reference proteins with the spliceosome or effects of downstream genes that are up- or downregulated and/or alternatively spliced. Interestingly, the alternatively spliced genes that are selectively associated with either CNS or RG promoter activation differ in their ontologies, even though nucleic acid binding proteins are markedly overrepresented in both groups (data not shown). In a previous study, ectopic expression of reference PGC-1α and various skeletal-muscle specific isoforms termed PGC-1α2, -α3 and α4 in mouse primary myotubes resulted in significant differences in the gene programs induced [50]. Noteworthy, the N-terminal protein domains accounted for the diversity of gene programs. The latter study revealed that transduction of PGC-1α isoforms was associated with pronounced differences in the splicing pattern of many genes including *Ddx27*, *Ndrg4,* and *Osbpl1a*. Our current data show that the human homologs of these genes are expressed in SH-SY5Y cells. While no alternative splicing of *OSBPL1A* was noted, activation of CNS and RG promoters displayed nearly identical effects on the splicing pattern of *NDRG4* and *DDX27* (Figures S17 and S18). These contrasting observations are in keeping with the specific effects of isoforms and tissue-specific effects [66].

Transcriptional co-regulators act at the amplification step of gene expression and may serve to integrate multiple transcriptional programs. Hence, only minor imbalances between co-regulatory expression or activity levels resulting from allelic variations, aberrant post-translational modification or altered generation of variant transcripts may contribute to the pathogenesis of various disease phenotypes. Sporadic forms of ALS, PD, and AD are thought to be multifactorial in that exogenous factors and a variable number of risk genes are thought to contribute to their pathophysiology. However, the overlap of risk genes among AD, PD, and ALS is low, while several studies suggest a modulatory role of PGC-1α isoforms in all three diseases. Hence, it is conceivable that the pathways or interactions whereby PGC-1α and its isoforms contribute to these disorders are distinct.

Our studies in a human cell line may not reflect the in vivo conditions in humans, and relationships to diseases are based on associations which do not imply causality. Nevertheless, previously established links of *PPARGC1A* with neurodegenerative disorders are supported by our current findings. DEGs resulting from both CNS and RG promoter activation showed associations with the ingenuity neurological disease classes Huntingon’s disease and chorea. CAG-expansions of *HTT* are thought to confer toxic gains of function [36] and clinical trials of lowering mutant huntingtin by antisense oligonucleotides are in progress [67]. Our studies also show that wild-type *HTT,* known to be important for multiple cellular functions including synaptic homeostasis [68,69], is upregulated by activation of the RG promoter. Mutant huntingtin represses transcription from the RG promoter by interfering with the CREB/TAF4-dependent transcriptional pathway, while ectopic expression of PGC-1α in the striatum provided neuroprotection in transgenic HD mice [15]. Whether CNS promoter activity is also repressed by mutant huntingtin, remains to be determined.

A role for PGC-1α in ALS has been suggested by several previous studies [19,70,71] and bioinformatics identified *PPARGC1A* as an upstream regulator of ALS related proteins [38]. Our enrichment analyses showed strong associations of CNS and, to a lesser extent, RG promoter activation with ingenuity classes neuronal development and functions. Furthermore, a large fraction of GWAS ALS genes was regulated by CNS promoter activation. The interpretation of the latter studies must be qualified, as the risk ratio differs for individual SNPs and genes and additional risk genes may be discovered. Nevertheless, one key example shows that *ANXA11* transcripts encoding Annexin A11 were strongly increased by both CNS and RG promoter activation (Table S5). As many proteins are translated in axons rather than in the cell soma, RNA must be transported for long distances. ANXA11 mediates the transport of, and tethers RNA granules to lysosomes via phase-separating and membrane binding domains [72,73]. Mutations in either domain impair the transport and can cause or increase the risk of ALS [73,74]. Sporadic ALS is characterized by large phenotypic and genetic heterogeneity, most likely reflecting a role of multiple cellular processes [39]. However, cytoplasmic aggregations of the RNA-binding protein TDP-43 (encoded by *TARDBP*) are present in the majority of ALS patients [75,76] and their propagation correlates with clinical symptoms and severity of the disease [77]. Aberrant location of TDP-43 in the cytoplasm results in its loss of function and impaired splicing repression in multiple genes including *STMN2*, an essential regulator of axon regeneration. Reduced binding of TDP-43 to *STMN2* intronic sequences generates short dysfunctional *STMN2* transcripts at the expense of full-length transcripts. Significantly, rescue of *STMN2* expression restored axonal regenerative capacity [42,78]. Our data show that activation of the CNS promoter upregulates full-length *STMN2* transcripts (Supplementary Material Tables S5 and S7), which might be beneficial for ALS, if alternative splicing activity becomes saturated.

In a meta-analysis of eight PD postmortem brain transcriptome studies, *STMN2* was also identified as a key regulator of functionally connected PD risk genes, and *Stmn2*-knockdown caused dopaminergic neuron degeneration [79]. Therefore, in addition to ALS, an increase in STMN2 transcription via CNS PGC-1α may also be beneficial for PD. Our current data reveal axon guidance signaling as the top canonical ingenuity signaling pathway in CNS promoter activated genes. Importantly, modeling of SNPs in axon guidance genes of a GWAS dataset predicted PD susceptibility (*p* = 4.64 × 10^−38^), survival free of PD (*p* = 5.43 × 10^−48^), and PD age of onset (*p* = 1.68 × 10^−51^) [80]. CNS and RG promoter activated genes showed strong associations with the ingenuity neurological disease ontology terms movement disorders and disorders of basal ganglia, and their respective negative z-values suggest a protective effect for these diseases. The central role of PGC-1α in mitochondrial biology and function also suggests a link to PD, even though the mitochondrial hypothesis for common neurodegenerative disorders has been challenged, as reduced oxidative phosphorylation and ATP synthesis repeatedly described in PD may be a secondary phenomenon [81]. However, mutations of *PINK1*, *PARKIN*, *DJ1*, and *VPS13C*, all involved in mitochondrial quality control or mitophagy, cause familial forms of PD. Furthermore, the production of new mitochondria at the periphery of the axonal tree depends on PGC-1α and is essential for axonal growth [82]. Thus, functions of CNS-specific isoforms and the reference protein may be complementary in several pathways. This hypothesis is supported by cross-talk shown to occur via co-activation of each other’s promoter [29]. Polygenic risk scores for sporadic PD, generated in large scale GWAS data from common variants of genes implicated in mitochondrial function, showed small, but additive effects on PD risk [83]. Furthermore, Mendelian randomization indicated potentially causal associations of 14 novel mitochondrial function genes with PD risk. Some of the newly identified genes were linked to known PD pathways such as lysosomal dysfunction and autophagy, while others pointed towards a novel role of mitochondrial ribosomes. Interestingly, our analyses of mitochondrial genes revealed enrichment of RG promoter activated genes involved in mitochondrial translation initiation, elongation, and termination. These results support common and distinct roles of CNS-specific and RG *PPARGC1A* isoforms in mitochondrial biology with relevance to PD.

Associations of *PPARGC1A* with cognitive decline [84], schizophrenia, [85], tremor [86], sense of smell [87], and AD [22] have been described in GWAS. Enrichment analyses disclosed a link of CNS promoter activation with several neuronal signaling pathways and ontologies of neuronal diseases such as cognitive impairment, all of which are relevant for AD (Figure 6b). A recent study showed that the deletion of synaptotagmin-2 (Syt2) from excitatory parvalbumin positive neurons in cerebellar nuclei produced asynchronous transmitter release and action tremor in mice [88]. Pgc-1α has been implicated in the transcriptional regulation of synchronous neurotransmitter release and *SYT2* expression [89]. We have previously observed a high expression level CNS-specific *Ppargc1a* transcripts in the cerebellum of mice [23] and our current data show an upregulation of *SYT2* (log2-fold change 0.589, adjusted *p* = 2.90 × 10^−6^) by activation of the CNS promoter, but no effects of RG promoter activation. Hence, CNS-specific isoforms may play a role in tremor disorders.

## 4. Material and Methods

### 4.1. Cell Culture

SH-SY5Y cells were obtained from ATCC and cultured in DME (D6046)/F12 1:1 (Sigma-Aldrich, Munich, Germany) supplemented with 0.21% NaHCO_3_. HEK293T cells, obtained from ATCC were cultured in Dulbecco’s modified Eagle’s medium/Ham’s F-12F (Merck, Kenilworth, NJ, USA). All media were supplemented with 10% fetal bovine serum (FBS, Gibco, Carlsbad, CA, USA) and 1% penicillin/streptomycin (Sigma-Aldrich). Cell densities of 80 to 90% were used to split and harvest cells using Accutase^R^ (Sigma-Aldrich).

### 4.2. Generation of Stable CRISPRa Cell Lines

For the stable integration of dCas9-VPR into the genome of cell lines, pLenti-EF1a-dCas9-SAM plasmids (ABM) along with the trans-complementation plasmids pMD2.G expressing VSV-G envelope and phCMV-8.91 expressing GAG/POL were transfected into HEK293T cells using jetPEI or Xfect transfection reagents (Polyplus Transfection^T^ or Clontech, Mountain View, CA, USA) in serum and antibiotic free medium. Virus-containing supernatants were harvested up to 72 h post transfection and cleared by centrifugation and filtration through 0.22 µm sterile filters. SH-SY5Y cells at a confluency of 60 to 70% were spinoculated at 600× *g* for 90 min at 37 °C with a range of virus concentrations in the presence of polybrene (5 µg/mL) and subsequently incubated for 8 h. Cells were washed with PBS and incubated with complete medium. dCas9-VPR positive cells were selected by culture with puromycin (5 µg/mL) for 14 days. After validation of dCas9-VPR transcript expression in cells, single cells were sorted into 96-well plates using the BD FACS Aria III system (BD Biosciences, Franklin Lakes, NY, USA). At confluency, single cell derived clones were split into duplicate 96-well plates. Cells from one plate were lysed using the ExCellent Lysis Kit (ABM, New York, NY, USA) and cDNA was generated from lysates using the all in-one RT master mix (ABM). Quantitative real-time PCR (qRT-PCR) was performed to discriminate between dCas9 positive and negative clones using GoTaq qPCR Master Mix (Promega, Madison, WI, USA) and primers designed for the detection of dCas9-VPR transcripts (Table S8).

### 4.3. Single Guide (sg)RNA Design and Cloning

To target the dCas9-VPR protein to the CNS and RG promoters, single guide (sg) RNAs were selected from the track “CRISPR Targets” embedded in the UCSC Genome Browser [90]. This track shows potential sgRNAs within 10,000 bp of transcribed regions in the human genome (hg 19) that are color coded according to predicted specificity (uniqueness in the genome) and efficiency (on-target cleavage) by algorithms such as the MIT Specificity Score [91] and the Doench Score [92] using the tool CRISPOR [93]. Three sgRNA target sequences predicted with high MIT-Specificity and Doench Scores as well as high specificity scores predicted by an additional web-based tool, CRISPRspec [94], were chosen within 260 bp of the transcription start sites of both promoters (Table S1). Regarding the CRISPRa system, the specificity scores are most relevant since the sgRNAs guide the dCas9 enzyme to the appropriate targets, but there is no cleavage. Nevertheless, we took all scores into account for selecting sgRNAs. Potential off-targets incorporating up to three mismatches as well as their associations with DEGs are shown in Table S2a,b.

pCRISPR-SG01 vectors expressing the designed sequences were obtained from GeneCopoeia. To estimate transient transfection efficiencies of sgRNAs, the gene encoding the enhanced green fluorescence protein (EGFP) was excised from the pEGFP-N1 vector (Clontech) and cloned into the sgRNA expressing plasmids. Enzyme sites KasI and NaeI (or BstZ17I) were chosen to replace the SV40 promoter and the hygromycin resistance cassette in the sgRNA plasmids with the cytomegalovirus promoter and the EGFP encoding gene. All plasmids were verified by sequencing (Microsynth).

### 4.4. Transient Transfections

SH-SY5Y cells were transfected with plasmids expressing sgRNA and/or EGFP using DNAfectin Plus (ABM) or Lipofectamine LTX with Plus reagent (Thermo Fisher, Waltham, MA, USA). Cells were plated in 6- or 24-well plates and transfected after reaching 70% confluency. Optimal transfection conditions were identified and used in subsequent experiments.

### 4.5. Confocal Microscopy and Immunocytochemistry

To determine the proportion of cells expressing EGFP after transient transfection with plasmids expressing both sgRNAs and EGFP, cells were detached using Accutase^R^ solution (Sigma-Aldrich) 24 h after transfection and were seeded on 30 mm borosilicate glass slides (thickness No. 1.0 VWR) coated with poly-L-lysine hydrobromide (Sigma-Aldrich). After 16 h, the cells were washed repeatedly with Hank’s balanced salt solution (HBSS, Sigma Aldrich), incubated with Hoechst 34580 (1 µg/mL, Sigma-Aldrich) for 30 min at room temperature, washed again with HBSS and transferred to the POCmini-2 chamber system (LaCon, Erbach-Bach, Baden-Württemberg, Germany). Images were collected using a Leica TCS SP5II AOBS confocal microscope (Leica Microsystems, Wetzlar, Germany) equipped with an HCX PL APO 63x/1.20 lambda blue water immersion objective and controlled with the Leica Application Suite Advanced Fluorescence (LAS AF) software. Hoechst 34580 was excited with a diode laser (405 nm) and emission was detected between 430 and 470 nm. EGFP was excited with the 488 nm line of the Argon laser and emission was detected in the 500 to 560 nm range.

To localize PGC-1α and estimate its cellular amount, we tested several antibodies directed against epitopes near the N-terminus. We interrogated their selectivity in transient transfections of SH-SY5Y cells with plasmids encoding EGFP fused to C-terminus of various PGC-1α isoforms. Based on these experiments, we selected a monoclonal anti-PGC-1α antibody, directed against N-terminal epitopes (Calbiochem, #ST1202). Transfected cells were transferred to round glass slides (12 mm diameter, thickness No. 1.0, VWR), grown overnight, fixed with 4% paraformaldehyde for 30 min at room temperature, permeabilized with 0.2% Triton X-100, blocked in 3% bovine serum albumin (BSA) for 1 h and incubated with the primary antibody diluted with HBSS and 0.1% BSA overnight at 4 °C. Cells were washed three times with HBSS and incubated with the secondary antibody (Goat Anti-Mouse IgG, Cy5 conjugate, Millipore) diluted 1:400 with HBSS and 0.1% BSA for 1 h at room temperature. Nuclei were counterstained with 0.1 µg/mL 4′6-diaminidino-2phenylindole (DAPI, Sigma-Aldrich) and slides were mounted in Mowiol/DAPCO. Images were collected by sequential acquisition with a Leica TCS SP5II AOBS confocal microscope (Leica Microsystems) equipped with an HCX PL APO 63x/1.4 oil immersion objective and controlled by the LAS AF SP5 software (Leica Microsystems). DAPI was excited with a diode laser (405 nm) and emission was detected between 430 and 480 nm. EGFP was excited with the 488 nm line of the Argon laser and emission was detected in the 500 to 560 nm range. Cy5 was excited with the HeNe laser (633 nm) and emission was detected in the 645 to 780 nm range. Samples were imaged using 3x zoom and a line average of 3.

### 4.6. RNA Extraction and qRT-PCR

QIAZOL Lysis Reagent and RNeasy Lipid Tissue Mini Kit (QIAGEN, Hilden, Germany) were used for extraction of RNA [23]. RNA concentration and integrity were measured using a Nanovue Spectrophotometer (GE Healthcare) and the QIAxcel Advanced Instrument (QIAGEN), respectively. DNase I-treated total RNA (1 μg/reaction) was reverse transcribed with the QuantiTect Reverse Transcription (RT) kit (QIAGEN), using a mix of random hexamer and oligo-dT primers, as described [95]. cDNAs were amplified in duplicate by real-time PCR using Maxima SYBR Green (Thermo Scientific) or GoTaq™ qPCR Master Mix (Promega), the LightCyclerTM480 (Roche) or Rotor-GeneTMQ (QIAGEN) instruments. For transcripts encoding PGC-1α isoforms, we used primers targeting exons B1 and B4 or B5 and exon 2, to quantify the two main CNS-specific *PPARGC1A* transcripts. RG transcripts were quantified using primers targeting exon 1 and exon 2. Primers targeting exon 5 and exon 7A or exon 2 and the extended part of exon 3 were used to measure the transcripts encoding the class of NT-PGC-1α isoforms or a short, dominant negative isoform termed E3extended, respectively (Table S1 for primer sequences). To directly compare measurements of *PPARGC1A* transcripts, gene segments containing the sequences targeted by the respective transcript-specific assays were cloned and used for the construction of standard curves. The accuracy of the assays was verified by sequencing amplicons. Primers used to estimate RNA levels of genes encoding other transcripts are shown in Table S8. Relative mRNA levels were calculated using the comparative threshold cycle method (Δ_CT_). Constitutively expressed RPLP0 (Ribosomal Protein, large, P0) RNA was used for normalization of mRNA abundance, as described [95]. Changes in *RPLP0* transcripts resulting from activation of the CNS or RG promoters were taken into account.

### 4.7. Cell Viability Assay

To quantify viable cells, the 3-(4,5-dimethylthiazol-2-yl)-2,5-diphenyltetrazolium bromide (MTT, Sigma-Aldrich) assay was used according to the protocol of the manufacturer.

### 4.8. RNA Sequencing

Sequencing libraries of three biological replicates were prepared using NEBNext Ultra II Directional Kit (New England Biolabs, Ipswich, MA, USA) In brief, 200 to 300 ng total RNA was used as input in the polyA enrichment module protocol. Enriched samples were fragmented and transcribed into cDNA. Following universal adapter ligation, samples were barcoded using dual indexing primers. Samples were sequenced to 30 to 50 million single-end 75 bp reads on the Illumina Nextseq 550 sequencer (Illumina).

### 4.9. RNA-Seq Bioinformatic Analysis

The raw sequencing reads were preprocessed with Trimmomatic (v 0.36) [96]. Briefly, adapters (ILLUMINACLIP: AGATCGGAAGAGCACACGTCTGAACTCCAGTCAC-:2:30:10:3) and low-quality (leading and trailing bases with Phred quality < 3 with subsequent sliding window of four consecutive bases with average Phred quality < 5 from the 3′) ends were trimmed. Reads shorter than 35 bp after the preprocessing were discarded. Quality of the reads was continuously monitored with FastQC (v0.11.5) [97]. CNS-specific isoforms of the *PPARGC1A* gene (RefSeq ID NM_001330751, NM_001330752, NM_001354825) [98] not present in the Ensembl gene annotation (release 91) [99] were added to the gene annotation used for genome mapping and expression estimates. The preprocessed reads were mapped to the reference human genome and transcriptome (GRCh38) with the extended gene annotation using STAR (v2.5.3a) [96]. Samtools (v1.4.1) was used to post-process the alignments [100]. Read coverage tracks were created by STAR. Expression estimates were calculated by RSEM (v1.3.1) [101]. Strand specificity of the library (reverse/antisense) was taken into consideration. DGE of coding genes was calculated by edgeR (v 3.26.8) [102] package in R (v3.6.2)/Bioconductor (v3.9) [103] using GLM [104]. The Benjamini-Hochberg procedure was used to correct *p*-values for multiple testing errors [105]. Additional visualizations were done by ggplot2 (v3.2.0; [106], dplyr (v0.8.2) ggrepel (v0.8.1), and ggpubr (v0.2.1) R libraries [107]. Alternative exon usage was calculated by DEXSeq (v1.30.0) [51]. Subread_to_DEXSeq script using featureCounts (v1.5.2) [108] was used to get exonic reads for the DEXSeq calculation. *p*-values were adjusted for multiple testing error with the default DEXSeq method. In addition, Sashimi plots for quantitative visualization and the MISO algorithm for estimation of isoform expression were used [109,110]. Sashimi plots were visualized with Gviz (v1.32.0) [111].

RNA-Seq data were analyzed using ingenuity pathway analysis (IPA, QIAGEN Inc.). Associations of differentially expressed mRNAs in our data sets with canonical pathways were determined in two ways: (i) the ratio of the number of mRNAs that map to the pathway divided by the total number of mRNAs that map to the canonical pathway is displayed; and (ii) A right-tailed Fisher’s Exact Test was used to calculate a *p*-value to determine the probability that the associations between the genes in the datasets and the canonical pathway is explained by chance alone. *p*-values were corrected by the Benjamini-Hochberg approach. We also determined the associations of mRNA from our datasets with physiological functions and/or diseases in the ingenuity knowledge base using the procedures described and provide z-scores for individual annotations when available. The *z*-scores are statistical measures of how closely the actual expression patterns compare to the patterns that are expected based on the literature/ingenuity knowledge base for a particular annotation [112]. Pathways, physiological functions and diseases clearly related to cancers were excluded from our results. To identify overrepresented mRNAs encoding mitochondrial proteins, we used the genes listed in Mitocarta2.0 [113]. To determine their biological functions, we used the Reactome pathway in The Gene Ontology (GO) Knowledge based platform (released 24 March, 2020) [114,115]. For in silico analysis of physical interactions of proteins encoded by the different gene sets, the search tool for retrieval of interacting genes the STRING data base [36] was used. Active physical interaction sources including text mining, experiments, and data base as well as a high confidence interaction score (>0.7) were used to construct the protein–protein networks. For visualization of networks, disconnected nodes were hidden.

## 5. Conclusions

Selective activation of the CNS and RG promoters in a human cell model is associated with discrete gene programs that include differential expression and exon usage of a multitude of genes with partial overlap. The respective gene programs reveal associations with qualitative and quantitative differences in canonical signaling pathways, physiological functions of the nervous system and neurological diseases and strongly support selective as well as complementary and common roles of the reference protein and CNS-specific isoforms in neurodegenerative disorders. Mining of these novel PGC-1α isoform targets should provide an invaluable resource for many research groups examining the intricate and complicated function of this multifaceted coactivator in neurodegenerative diseases.

## Figures and Tables

**Table 1:** Associations between risk genes of neurodegenerative diseases and differentially expressed genes induced by CNS or RG promoter activation.

Disease	Risk Genes	CNSPA Genes ^1^	*p*	RGPA Genes ^2^	*p*
Amyotrophic lateral sclerosis	58	36	0.0013	20	0.0171
Alzheimer’s disease	63	29	0.0311	15	0.1892
Parkinson’s disease	141	55	0.0456	48	<0.0001

Expression changes: adjusted *p*-value < 0.05 and fold change-ratio > 1.05; ^1^ CNS promoter activated, ^2^ RG promoter activated; 8, 14, or 26 risk genes of ALS, AD, or PD, respectively, were not or at a very low level expressed in SH-SY5Y cells.

## Data Availability

Data used and analyzed for the current study are available from the corresponding author on reasonable request.

## Supplementary Materials

The following are available online at https://www.mdpi.com/1422-0067/22/7/3296/s1, Figure S1: mRNA expression levels of dCas9-VPR in clonal SH-SY5Y cell lines, Figure S2: Genomic PPARGC1A locus, Figure S3: Fluorescent imaging of SH-SY5Y dCAS9-VPR positive clonal cells, Figure S4: Specificity of the antibody raised against the N-terminal (Nt) region of reference PGC1-α, Figure S5: Metabolic activity of clonal dCAS9-VPR expressing SH-SY5Y cells, Figure S6: Multidimensional Scaling (MDS) plot, Figure S7: Sashimi plots of RG and CNS transcripts, Figure S8: Expression and exon usage of RG and CNS exon bins in cells transfected with sgRNAs targeting the respective promoter, Figure S9: MA plots of differentially expressed genes (DEGs) in cells with RG or CNS activated promoters, Figure S10: Verification of selected DEGs in clonal dCAS9-VPR—expressing SH-SY5Y cells after activation of RG or CNS promoters, Figure S11: Predicted physical interactions of proteins translated from all genes upregulated after activation of the RG promoter, Figure S12: Predicted physical interactions of proteins translated from all genes upregulated after activation of the CNS promoter, Figure S13: Predicted physical interactions of proteins translated from all genes selectively upregulated after activation of the RG promoter, Figure S14: Predicted physical interactions of proteins translated from all genes selectively upregulated after activation of the CNS promoter, Figure S15: Exon usage of RPS27A, Figure S16: Exon usage of RPL12, Figure S17: Exon usage of NDRG4, Figure S18: Exon usage of DDX27, Table S1: Specificities and efficiency scores of sgRNAs targeting the reference gene and the CNS-specific promoters, Table S2: Effects of mismatches in sgRNAs targeting the CNS reference gene promoter on in silico off-targets, Table S3: Activation of CNS or reference PPARGC1A gene promoters and differential expression of genes encoded by mitochondria, Table S4: Predicted physical protein-protein interactions and physical Local Network Clusters (STRING), Table S5: Changes in expression levels of ALS associated genes by activation of the PPARGC1A CNS or reference gene promoters, Table S6: Changes in expression levels of AD associated genes by activation of the PPARGC1A CNS or reference gene promoters, Table S7: Changes in expression levels of PD associated genes by activation of the PPARGC1A CNS or reference gene promoters, Table S8: Primers for RT-PCR studies.

## Abbreviations

AD	Alzheimer’s disease
ALS	amyotrophic lateral sclerosis
CRISPR	clustered regularly interspaced short palindromic repeats
CRISPRa	CRISPR activating transcription
DEG	differentially expressed genes
EGFP	enhanced green fluorescence protein
HD	Huntington’s disease
MDS	multidimensional scaling
Mito-genes	mitochondrial genes
MT-genes	genes encoded by mitochondria
MTT	3-(4,5-dimethylthiazol-2-yl)-2,5-diphenyltetrazolium bromide
NT-PGC-1α	N-terminal truncated PGC-1α
PD	Parkinson’s disease
PGC-1α	peroxisome proliferator activated receptor gamma coactivator 1A
qRT-PCR	quantitative real-time PCR
RG	reference gene
RRM	RNA recognition motif
RS domain	arginine/serine-rich domain
sgRNA	single guide RNA
uORF	upstream open reading frame
VPR	tripartite activator called VPR encoding herpes simplex virus protein 16 (VP64), the activation domain of the p65 subunit of nuclear factor NF-κB and the replication and transcription activator (RTA) of the γ-herpesvirus family
