# Supplementary material for: Selective Activation of CNS and Reference PPARGC1A Promoters Is Associated with Distinct Gene Programs Relevant for Neurodegenerative Diseases
Source: Int J Mol Sci. 2021 Mar 24;22(7):3296. doi: 10.3390/ijms22073296 (PMC8036390; doi:10.3390/ijms22073296)
Supplement: Supplementary file 1 [file ijms-22-03296-s001.pdf]

Supplementary Tables

Table S1 Specificities and efficiency scores of sgRNAs targeting the reference gene and the CNS-specific promoters

**sgRNA**	**Sequence 5‘to 3‘**	**PAM**[a]	**Position**[b]	**MIT Guide Specificity Score**	**CRISPspec Score**	**Doench Efficiency Score (%)**	**Potential Off-Targets For Number of Mismatches**			
							0	1	2	3
**RG1**	GCGTTACTTC ACTGAAGCAG	AGG	-258 to -239	82	7.394	86	0	0	0	13
**RG2**	TAGAGCAGCA AGCTGCACAG	GGG	-210 to -191	68	7.464	98	0	0	3	14
**RG3**	ACTGGGGACT GTAGTAAGAC	AGG	-132 to -112	85	8.321	86	0	0	0	5
**CNS1**	AGATAATGAG CGAGCCGGGA	TGG	-69 to -82	89	8.362	63	0	0	0	4
**CNS2**	CGCAGAGCGA GAGACCGCAG	GGG	-34 to -56	83	8.320	94	0	0	0	8
**CNS3**	CGCACACACG CAGCCGGCAC	AGG	+5 to +24	83	8.030	56	0	0	1	7

*Protospacer Adjacent Motif; RG1-3, reference gene promoter-specific sgRNAs; CNS1-3, CNS-promoter specific sgRNAs. The MIT specificity and the Doench efficienty scores ranges from 0-100 and summarizes off-target effects into a single number; the higher the number the fewer off target effects are anticipated. The CRISPRspec score represents overall whole genome off-targeting; all scores were in the high range, indicating low off-target predictions (rth.dk/resources/crispr/crisproff); please see main text for additional references.

Table S2a Effects of mismatches in sgRNAs targeting the CNS promoter on *in silico* off-targets

sgRNA	Potential Off-target number	Locus, Position	CNS vs Scrambled*	RG vs scrambled*
**CNS 1**				
2 mismatches				
	0			
3 mismatches				
	4	Intergenic MIR5095-CTCFL; chr 20:56017262(-)	Not in file	Not in file
		Intron ASAP1; chr6:131204007(+)	0.145-fold down, *p*=0.007	0.353-fold down, *p* =2.4E-11
		Intergenic AK124832-CA10; chr 17:49479286(+)	Not in file	Not in file
		Intergenic AKAP7-ARG1; chr 6:131676375(+)	n.s.; not in file	n.s.; not in file
**CNS 2**				
2 mismatches				
	0			
3 mismatches				
	8	Intergenic LYRM2-ANKRD6;chr 6:90300681(-)	n.s.; n.s.	n.s.; n.s.
		Intergenic TPCN2/BC064339.MYEOV;chr 11:68881239(-)	n.s.; not in file	n.s.; not in file
		Intron MAD1L1; chr 7:2122602(-)	0.476-fold up, *p*=3-19E-12	0.460-fold up, *p* =4.08E-11
		Intergenic FBXO47-LRRC37A11P;chr17:37174551(+)	Not in file; n.s.	Not in file; n.s.
		Intron AACS;chr 12:125579071(+)	n.s.	n.s.
		Intergenic LOC100130700-RNA5SP411/FLJ26245;chr16:34778137(-)	Not in file	Not in file

		Intergenic NCOR2-SCARB1; chr 12:125090098(+)	0.387-fold up, *p* =1.95E-10; 0.424-fold up, *p*=1.4E-09	0.419-fold up, *p*=1.69E-11; 0.140-fold up, *p* =0.083
		Intron UMODL1; chr 21:43516739(-)	Not in file	Not in file
**CNS 3**				
2 mismatches				
	1	Intergenic ZBTB18/AK310634-C1orf100; chr 1:244388184(+)	n.s., not in file	0.132-fold-up, *p* =0.033, not in file
3 mismatches				
	7	Intron ACOT7;chr 1:6359884(+)	0.358-fold up, *p* =1.1E-09	0.246-fold up, *p* =8.90E-05
		Intron AYA4;chr 6:133563395(-)	n.s.	n.s.
		Intergenic LANCL3-XK;chr X:37544905(+)	Not in file	Not in file
		Intron FGFR3;chr 4:1796528(+)	0.0423-fold down, *p* =0.01	n.s.
		Intergenic LOC100506207-OFCC1/MRDS1;chr 6:9399387(-)	Not in file	Not in file
		Intron BAIAP2;chr 17:79042391(+)	n.s.	n.s.
		Intron AJAP1;chr 1:4715528(-)	Not in file	Not in file

*for intergenic regions, the first statement refers to the first gene mentioned and the second statement to the second gene mentioned; n.s., no significant change in expression.

Table S2b Effects of mismatches in sgRNAs targeting the reference gene promoter on *in silico* off-targets

sgRNA	Potential Off-target number	Locus, Position	RG vs scrambled*	CNS vs scrambled*
**RG 1**				
2 mismatches				
	0			
3 mismatches				
	13	Intron MFSD10;chr 4:2933484	n.s.	0.248-fold up, *p*=0.0001
		Intergenic PCP4-DSCAM;chr 21:41373320(+)	Not in file	Not in file
		Intergenic VPS37C-PGA3;chr 11: 609363324	n.s.; not in file	n.s.; not in file
		Intergenic LINC00900-Mir_652;chr 11:115834494	Not in file	Not in file
		Intergenic L37717-ZNF733P, CHR 7:62709008(-)	Not in file	Not in file
		Intergenic SEPT7P9;chr 10:38688991	Not in file	Not in file
		Intergenic SEPT7P2;chr 7:45805745	Not in file	Not in file
		Intergenic DL490813-ZNF479; chr. 7:57142701(-)	Not in file	Not in file
		Intergenic LOC100287834-MIR4283-1;chr 7:62955844(+)	Not in file	Not in file
		Intergenic DQ599872-AK124970;chr 1:224183241(+)	Not in file	Not in file
		Intron LOC645513;chr 4:120378771(+)	Not in file	Not in file
		Intergenic LOC100288069-LINC00115;chr 1:716961	Not in file	Not in file
		Intergenic DQ599872-AK124970;chr 1224183241(+)	Not in file	Not in file
		Intron LOC645513;chr 4:120378771(+)	Not in file	Not in file
		Intergenic LOC100288069-LINC00115;chr 1:716961(+)	Not in file	Not in file
		Intergenic GABARAPL1-KLRD1;chr 12:10400702(-)	n.s.; not in file	0.599-fold down, *p*= 4.6E-07; not in file

**RG 2**				
2 mismatches				
	3	Intergenic TNS4-CCR7;chr 17:38662563(-)	Not in file	
		Intergenic SMARCA4-LDLR;chr 19:11178504(+)	n.s.; 0.219-fold up, *p* =0.014	0.314-fold up, *p* =1.7E-09; 0.214-fold up *p*=0.01
		Intron SCAI;chr 9:127874179(+)	n.s.	n.s.
3 mismatches				
	14	Intergenic ZCAH2-ZC3H12B;chr X:64689095(+)	n.s.; n.s.	n.s.; n.s.
		Intergenic U6atac-AK128059;chr 11:131260302(+)	Not in file	Not in file
		Intron WBSCR17;chr 7:70634123(-)	Not in file	Not in file
		Intergenic CALN-TYW1B;chr 771949606(-)	Not in file; n.s.	Not in file, n.s.
		Intron FAM185A;chr 7102447374(+)	n.s.	n.s.
		Intron TNRC6B;chr 22:40443758(-)	n.s.	0.163-fold up, *p*=0.044
		Intergenic FGFBP1-FGFBP2;chr 4:15953483(+)	Not in file	Not in file
		Intron OPCML;chr 11:132783443(+)	Not in file	Not in file
		Intergenic CYB561-ACE;chr 17:61528081	n.s.; n.s.	0.479-fold up, *p* =1.7E-08; 0.3-fold up, *p* =0.0016
		Intergenic LOC257396-FST;chr 5:52614293	Not in file	Not in file
		Intergenic HAND1-MIR3141;chr 5:153961950(+)	n.s.; not in file	0.434-fold up, *p* =6.6E-10; not in file
		Intergenic TGFB2-LOC643723;chr 1:218917293(-)	Not in file	Not in file
		Intergenic PTPRD-JB1753000;chr 9:11502106(+)	n.s.; not in file	n.s.; not in file
		Intron LOC285501;chr 4:178904870(+)	Not in file	Not in file
**RG 3**				

2 mismatches				
	0			
3 mismatches				
	5	Intergenic MFAP3L-BC031941;chr 4:170950596(-)	n.s.; not in file	n.s.; not in file
		Intron TNRC6B;chr 22:40529575(+)	n.s.	0.163-fold up, *p* =0.044
		Intron THSD7B;chr 2:137900825(-)	Not in file	Not in file
		Intergenic AF086294-FSTL4;chr 5:132460458(+)	Not in file	Not in file
		Intron SOX5;chr 12:23923679(+)	n.s.	n.s.

*for intergenic regions, the first statement refers to the first gene mentioned and the second statement to the second gene mentioned; n.s., no significant change in expression.

Table S3 Activation of CNS or reference *PPARGC1A* gene promoters and differential expression of genes encoded by mitochondria

**Gene**	**Mitochondrial complex**	**CNS *PPARGC1A***		**Reference *PPARGC1A***	
		**Log2-fold Change**	***p*-adj**	**Log2-fold Change**	***p*-adj**
**MT-ND1**	I	0.330	4.14E-06	0.123	1.02E-01
**MT-ND2**	I	0.318	1.02E-05	-0.063	4.95E-01
**MT-ND3**	I	0.206	1.86E-03	-0.114	1.31E-01
**MT-ND4**	I	0.293	2.44E-04	0.080	4.20E-01
**MT-ND4L**	I	0.599	3.33E-18	0.486	8.20E-12
**MT-ND5**	I	0.504	7.38E-10	0.230	1.11E-02
**MT-ND6**	I	1.192	9.89E-58	0.411	2.94E-07
**MT-CYB**	III	0.320	3.82E-06	0.112	1.68E-01
**MT-CO1**	IV	0.263	1.83E-05	0.104	1.42E-01
**MT-CO2**	IV	0.286	7.43E-04	0.104	3.06E-01
**MT-CO3**	IV	0.322	5.02E-06	0.031	7.58E-01
**MT-ATP6**	V	0.082	2.73E-01	-0.218	2.47E-03
**MT-ATP8**	V	-0.151	1.90E-02	-0.559	1.36E-19

Data show the differences in comparison between CNS or RG promoter activations with transfections of scrambled sgRNA.

Table S4 Predicted physical protein-protein interactions and physical Local Network Clusters (STRING)

Table S4a Predicted physical protein-protein interactions of proteins encoded by various genes sets

**Gene set**[1]	**Number of Genes**	**Protein-Protein Interactions**			**Average Local Clustering Coefficient (STRING)**
		Predicted	Expected	P^2	
**RGP all up**	872	517	449	0.0010	0.237
**CNSP all up**	1792	1834	1591	1.5E-09	0.218
**RGP selective up**	452	138	125	0.1300	0.173
**CNSP selective up**	1374	963	811	1.1e-07	0.203
**RGP up CNSP down**	29	18	8	0.0018	0.363
**RGP down CNSP up**	27	11	6	0.0392	0.262
**RGP up CNSP up**	391	120	94	0.0064	0.199

[1]For gene sets refer to Figure 4a; RGP, RG promoter; CNSP, CNS promoter; selectively upregulated genes without down- or upregulation by the activation of the other promoter; RGP up CNSP up refers to genes upregulated by both RG and CNS promoters.

[2] The smaller the value, the higher the probability that the proteins encoded by the respective gene set show more interactions among themselves than would be expected for a random set of proteins of similar size, drawn from the genome. Such enrichment indicates that the proteins are at least partially biologically connected, as a group.

Table S4b Predicted physical protein interactions of PPARGC1A in various gene sets

**Gene Set**		**Predicted Physical Interactions of PPARGC1A**
**RGP all up**	7	EP300, ESRRA, MED16, MED26, MED27, 29MED, POLR2
**CNSP all up**	6	ESRRA, HELZ2, MED15, MED16, MED25, RXRA
**RGP selective up**	4	EP300, MED26,MED27, MED29
**CNSP selective up**	4	HELZ2,MED15, MED25, RXR
**RGP up CNSP down**	1	ITPKB
**RGP down CNSP up**	3	ESRRA, MED1, PPARG
**RGP up CNSP up**	2	ESRRA, MED16

Interaction Score for all entries >0.85.

Table S4c Predicted physical Local Network Clusters (STRING)

**Gene Set**	**N**	**Local Network Cluster (STRING)**[1]
**RGP all up**	5	CL:22448, 7 of 23, cytochrome complex, and Cytochrome c oxidase subunit VII (1.12; 0.0049)<br>CL:21102, 5 of 20; tricarboxylic acid cycle (1.04; 0.0470)<br>CL:12805, 5 of 21, Activation of the pre-replicative complex (1.02; 0.049)<br>CL:21101, 7 of 34, tricarboxylic acid cycle, and lactate dehydrogenase activity (0.95; 0.0123)<br>CL:22328, 10 of 94, respirasome (0.67; 0.032)
**CNSP all up**	9	CL:403, 23 of 101, Axon guidance, and Hydantoinase/dihydropyrimidinase (0.51; 0,.0057)<br>CL:21009, 26 of 160, Carbon metabolism, and Amino sugar and nucleotide sugar metabolism (0.37; 0.380)<br>CL:587, 5 of 5; Hydantoinase/dihydropyrimidinase (1.16; 0.0418<br>CL:21008, 33 of 200, mixed, incl. Carbon metabolism, and Carbohydrate metabolism, (0.37; 0.0064)<br>CL:14976, 22 of 89, Peptide chain elongation (0.55; 0.0057)<br>CL:14978, 21 of 84, Peptide chain elongation (0.56; 0.0057)<br>CL:14982, 19 of 73, Peptide chain elongation (0.57; 0.0057)<br>CL:14983, 17 of 63, Peptide chain elongation (0.59; 0.0057)<br>CL:14985, 15 of 58; Viral mRNA Translation (0.57; 0.0146)
**RGP selective up**	8	CL:3141, 4 of 6, AXIN missense mutants destabilize the destruction complex (1.46; 0.0097)<br>CL:12835, 4 of 6, Magnesium chelatase, subunit ChlI (1.46; 0.0097)<br>CL:3139, 5 of 11, protein phosphatase type 2A complex (1.29; 0.0096)<br>CL:22448, 7 of 23, cytochrome complex, and Cytochrome c oxidase subunit VII (1.12, 0.0053)<br>CL:12805, 6 of 21, Activation of the pre-replicative complex (1.09; 0.0096)<br>CL:21102, 5 of 20, tricarboxylic acid cycle (1.03; 0.0247)<br>CL:21101, 7 oof 34, tricarboxylic acid cycle, and lactate dehydrogenase activity (0.95; 0.0096)<br>CL:22328, 10 of 94, respirasome (0.66; 0.0159)
**CNSP selective up**	8	CL:587, 5 of 5, Hydantoinase/dihydropyrimidinase (1.16; 0.0424)<br>CL:14983, 17 of 63, Peptide chain elongation (0.59; 0.0066)<br>CL:14982, 19 of 73, Peptide chain elongation (0.57; 0.0061)

		CL:14985, 15 of 58, Viral mRNA Translation (0.57; 0.0152)<br>CL:14976, 22 of 89, Peptide chain elongation (0.55; 0.0061)<br>CL:403, 22 of 101, Axon guidance, and Hydantoinase/dihydropyrimidinase (0.49; 0.0066)<br>CL:21008, 33 of 200, mixed, incl. Carbon metabolism, and Carbohydrate metabolism (0.37; 0.0068)<br>CL:21009, 29 of 160, Carbon metabolism, and Amino sugar and nucleotide sugar metabolism (0.37; 0.0401)
**RGP up CNSP down**	5	CL:18916, 3 of 5, HOPS complex (2.53; 4.90e-06)<br>CL:2482, 4 of 12, AP-1 transcription factor, and Transcription factor Jun (2.27; 6.87e-07)<br>CL:18913, 4 of 12, HOPS complex, and Mon1-Ccz1 complex (2.27; 6.87e-07)<br>CL:2484, 2 of 6, transcription factor AP-1 complex, and Fos-related antigen 2 (2.27; 0.0010)<br>CL:2481, 5 of 18, bZIP transcription factor, and Early growth response, N-terminal (2.19, 5.98e-08)
**RGP down CNSP up**	6	CL:15779, 2 of 5, Oestrogen-related receptor, and PGC-1 (2.45; 0.0046)<br>CL:329, 2 of 15 PDGF/VEGF domain, vascular endothelial growth factor-activated receptor activity (1.97, 0.01)<br>CL:6081, 2 of 21, Notch-HLH transcription pathway, and Competing endogenous RNAs (ceRNAs) regulate PTEN translation (1.82; 0.011)<br>CL:8754, 3 of 78, mostly uncharacterized, incl. Receptor-type tyrosine-protein phosphatases, and peptide hormone processing (1.43; 0.0013)<br>CL:11966, 2 of 34; mediator complex (1.61; 0.0134)<br>CL:5182, 3 of 87, mixed, incl. cell fate commitment, and Homeobox, conserved site (1.05; 0.021)
**RGP up and CNSP up**	0	

[1]Data show Cluster ID, number of proteins encoded by the respective gene set, number of all proteins in the respective network, name of the network, (strength; p-values corrected for false discovery).

Table S5 Changes in expression levels of ALS associated genes by activation of the *PPARGC1A* CNS or reference gene promoters

**Gene Name**	**CNS Promoter Activation**		**Reference Promoter Activation**		**References**
	**Log2-fold change**	***p*-adj**	**Log2-fold change**	***p*-adj**	
**Differentially Expressed Risk Genes**					
ALS2	-0.268	2.63E-05	-0.029	7.46E-01	1,2
ANXA11	0.636	3.11E-21	0.422	2.21E-09	2
ATXN2	-0.384	6.73E-12	0.284	1.34E-06	1,2
CHMP2B	-0.237	4.52E-03	-0.146	1.18E-01	1,2
CFAP410	0.264	1.74E-02	0.145	2.58E-01	2
C19orf12	0.293	1.25E-05	0.065	4.93E-01	1
CHCHD10	0.270	2.74E-03	0.317	7.61E-03	1,2
CHRNA3	0.334	4.67E-13	- 0.090	1.24E-01	1
CHRNB4	0.506	2.82E-18	0.262	8.68E-05	1
CRYZ	-0.294	1.40E-05	0.142	6.31E-02	3
DCTN1	0.263	7.67E-06	0.060	4.19E-01	1,2
DNAJC7	-0.216	5.08E-05	0.050	5.03E-01	4
ELP3	-0.216	9.79E-03	-0.005	9.71E-01	1,2
FGD4	0.402	1.10E-02	0.180	3.42E-01	3
FIG4	-0.286	2.52E-04	-0.214	1.13E-02	1,2
FUS	0.135	1.74E-02	0.079	2.26E-01	1,2
HNRNPA1	0.148	6.72E-03	0.168	3.55E-03	1,2
MATR3	-0.130	2.84E-02	-0.231	1.12E-04	1,2
NEFH	0.105	2.17E-01	-0.200	2.12E-02	1,2
NEK1	-0.291	3.27E-05	-0.247	9.70E-04	1,2
OPTN	-0.125	1.31E-01	-0.192	2.38E-02	1,2
PNPLA6	0.178	2.10E-03	0.156	2.53E-02	1
PON2	-0.258	1.48E-06	-0.275	5.60E-06	1
PRPH	0.234	4.38E-03	0.100	3.08E-01	1,[3]2
SARM1	0.323	4.38E-08	0.300	1.12E-05	5
SCFD1	-0.510	6.40E-12	0.080	4.27E-01	5
SETX	-0.273	5.23E-05	-0.152	4.35E-02	1,2
SIGMAR1	0.257	6.53E-05	0.194	5.35E-03	1,2
SPG11	-0.301	3.13E-06	0.071	3.76E-01	1,2
SQSTM1/p62	-0.445	7.94E-07	0.335	5.22E-04	1,2
STMN2	0.323	2.52E-05	-0.258	1.74E-03	6,7
SUSD2	-1.204	5.20E-08	0.409	1.63E-01	8
TAF15	0.175	1.05E-02	0.009	9.35E-01	1,2
TARDBP	-0.119	3.00E-02	0.107	7.31E-01	1,2
TBK1	-0.291	1.43E-05	-0.272	4.58E-04	1
TIA1	-0.173	8.65E-03	-0.315	1.99E-06	2
TYW3	-0.392	5.50E-10	-0.039	6.72E-01	3
VCP	0.140	3.84E-03	0.151	2.98E-03	1,2

**Risk Genes without Expression Change**					
C9orf72	-0.133	1.22E-01	-0.143	1.20E-01	1,2
ERBB4	0.035	9.48E-01	0.035	9.48E-01	1,2
ERLIN1	0.116	8.50E-01	0.030	7.35E-01	2
EWSR1	0.085	1.27E-01	0.037	5.83E-01	1
KIF5A	0.168	6.79E-01	0.023	8.54E-01	9
LMNB1	0.037	6.12E-01	0.071	3.42E-01	2
PFN1	0.087	1.38E-01	0.109	7.67E-02	1,2
SOD1	0.048	5.56E-01	0.142	6.78E-01	1,2
SPAST	0.023	7.85E-01	0.124	1.13E-01	2
SS18L1	0.069	3.49E-01	0.033	7.14E-01	1
UBQLN2	0.112	7.75E-01	0.011	9.02E-01	1,2
UNC13A	0.102	2.66E-01	0.069	5.14E-01	2
**Risk Genes not Expressed in SHSY5Y Cells**					
ANG					1,2
CHRNA4					1
DAO					1,2
HNRNPA2B1					1
PON1					1
PON3					1
TUBA4A					1,2
VAPB					1,2

p, adjusted by Benjamini Hochberg procedure; upregulation or repression relative to transfection with scrambled sgRNA are highlighted in green or red, respectively.

Reference List

1. Mejzini R, Flynn LL, Pitout IL, Fletcher S, Wilton SD, Akkari PA. ALS Genetics, Mechanisms, and Therapeutics: Where Are We Now? Front Neurosci 2019;13:1310.

2. Dervishi I, Gozutok O, Murnan K et al. Protein-protein interactions reveal key canonical pathways, upstream regulators, interactome domains, and novel targets in ALS. Sci Rep 2018;8(1):14732.

3. Wei L, Tian Y, Chen Y et al. Identification of TYW3/CRYZ and FGD4 as susceptibility genes for amyotrophic lateral sclerosis. Neurol Genet 2019;5(6):e375.

4. Farhan SMK, Howrigan DP, Abbott LE et al. Exome sequencing in amyotrophic lateral sclerosis implicates a novel gene, DNAJC7, encoding a heat-shock protein. Nat Neurosci 2019;22(12):1966-1974.

5. van Reenen W, Shatunov A, Dekker AM et al. Genome-wide association analyses identify new risk variants and the genetic architecture of amyotrophic lateral sclerosis. Nat Genet 2016;48(9):1043-1048.

6. Klim JR, Williams LA, Limone F et al. ALS-implicated protein TDP-43 sustains levels of STMN2, a mediator of motor neuron growth and repair. Nat Neurosci 2019;22(2):167-179.

7. Melamed Z, Lopez-Erauskin J, Baughn MW et al. Premature polyadenylation-mediated loss of stathmin-2 is a hallmark of TDP-43-dependent neurodegeneration. Nat Neurosci 2019;22(2):180-190.

8. Deng M, Wei L, Zuo X et al. Genome-wide association analyses in Han Chinese identify two new susceptibility loci for amyotrophic lateral sclerosis. Nat Genet 2013;45(6):697-700.

9. Nicolas A, Kenna KP, Renton AE et al. Genome-wide Analyses Identify KIF5A as a Novel ALS Gene. Neuron 2018;97(6):1268-1283.

Table S6 Changes in expression levels of AD associated genes by activation of the *PPARGC1A* CNS or reference gene promoters

Gene Name	CNS Promoter Activation		Reference Promoter Activation		References
	Log2-fold change	*p*-adj	Log2-fold change	*p*-adj	
**Differentially Expressed Risk Genes**					
ACE	0.300	1.61E-03	0.116	3.09E-01	1,2,3
ADAM10	-0.185	1.41E-04	-0.137	3.06E-02	1,2,3
ADAMTS1	0.130	4.63E-02	0.109	1.30E-01	1,2
ADAMTS4	0.527	5.04E-14	0.368	6.41E-07	2,3
APOE*	1.940	1.00E-5	2.870	1.00E-5	1,2,3
APP	0.257	2.58E-07	-0.009	9.08E-01	3
BIN1	0.294	1.20E-07	0.087	1.89E-01	1,2,3
CD2AP	-0.414	3.20E-09	-0.438	1.31E-09	1,2,3
CLU	-0.003	9.66E-01	0.262	4.30E-05	1,2,3
CNTNAP2	-0.652	5.18E-08	-0.358	6.14E-03	2,3
DEDD	0.219	3.20E-04	0.238	1.92E-04	2
ECHDC3	0.301	2.45E-02	0.074	6.73E-01	1,2,3
ENAH	-0.269	3.13E-07	-0.094	1.21E-01	2
FERMT2	-0.282	2.67E-08	0.103	7.45E-02	1,3
HBEGF	-0.801	3.38E-09	0.005	9.82E-01	3
INPP5D	-0.351	2.02E-02	-0.399	1.22E-02	1,2,3
IQCK	-0.316	7.37E-03	-0.038	8.17E-01	1,3
KANSL1	0.167	1.97E-02	0.267	6.75E-03	3
KAT8	0.179	8.76E-03	0.236	8.46E-04	2,3
MAPT	0.738	4.56E-08	0.199	2.21E-01	3
NDUFAF7	-0.260	4.21E-03	0.154	1.31E-01	3
NYAP1	0.286	7.42E-04	0.206	2.67E-02	1
PICALM	-0.419	9.51E-08	-0.071	4.81E-01	1,2,3
RAB8B	-0.263	1.66E-02	-0.248	3.39E-02	2
SORL1	0.284	1.55E-04	0.003	9.77E-01	1,2,3
TP53INP1	-0.080	1.98E-01	-0.203	8.98E-04	3
TRIP4	-0.367	1.38E-05	-0.014	9.07E-01	3
WDR12	-0.193	1.59E-03	-0.176	7.29E-03	2
WIPI2	0.122	1.98E-02	0.009	9.04E-01	2
ZCWPW1	-0.607	2.01E-05	0.058	7.62E-01	2,3
ZNF423	0.710	6.52E-19	0.334	1.10E-04	2
**Risk Genes without expression change** C					
ABCA7	-0.147	3.31E-01	-0.033	8.69E-01	1,2,3
APH1B	-0.037	6.49E-01	-0.002	9.88E-01	2,3
c14orf93	0.088	4.32E-01	0.066	6.08E-01	2
C17orf107	0.139	7.39E-01	0.070	8.92E-01	3
CCDC6	0.005	7.40E-01	0.027	7.45E-01	2

DSG2	-0.533	8.24E-02	0.188	6.06E-01	3
HESX1	-0.593	8.00E-02	-0.571	1.19E-01	2,3
MEF2C	-0.036	8.28E-01	0.177	2.55E-01	3
PLCH1	-0.029	8.75E-01	-0.237	1.69E-01	2
PRKD3	-0.07	3.34E-01	-0.153	5.29E-02	3
PTK2B	-0.121	3.92E-01	0.233	9.99E-02	1,2,3
RORA	-0.075	7.76E-01	-0.003	9.92E-01	3
SHARPIN	0.025	8.32E-01	0.178	9.52E-02	3
SPPL2A	-0.004	9.65E-01	-0.117	1.02E-01	3
TPBG	-0.015	8.46E-01	-0.006	9.49E-01	3
WWOX	0.027	9.03E-01	-0.149	5.22E-01	1
ZNF652	0.098	3.92E-01	0.124	3.12E-01	2
ZNF655	-0.114	5.46E-02	-0.093	1.55E-01	3
**Risk Genes not Expressed in SHSY5Y Cells**					
CR1					1
HLA-DQB1					1
HLA-DRB1					1,2,3
TREM2					1,2,3
EPHA1					1,2,3
SPI1					1,2,3
MS4A2					1,2,3
SLC24A4					1,2,3
SCIMP					2,3
ABI3					2,3
CD33					2,3
CASS4					1,2,3
AC074212.3					2
CLNK					2,3
APKL2					2,3
PRL					2
TMEM184A					2
IL34					2,3
PLCG2					3
NME8					3
IGHV1-67					3
IGHG3					3
TREML2					3
CHRNE					3

p, adjusted by Benjamini-Hochberg procedure; upregulation or repression relative to transfection with scrambled sgRNA are highlighted in green or red, respectively.

Reference List

1. Kunkle BW, Grenier-Boley B, Sims R et al. Genetic meta-analysis of diagnosed Alzheimer's disease identifies new risk loci and implicates Abeta, tau, immunity and lipid processing. Nat Genet 2019;51(3):414-430.

2. Jansen IE, Savage JE, Watanabe K et al. Genome-wide meta-analysis identifies new loci and functional pathways influencing Alzheimer's disease risk. Nat Genet 2019;51(3):404-413.

3. Sims R, Hill M, Williams J. The multiplex model of the genetics of Alzheimer's disease. Nat Neurosci 2020;23(3):311-322.

Table S7 Changes in expression levels of PD associated genes by activation of the *PPARGC1A* CNS or reference gene promoters

Gene Name	CNS Promoter Activation		Reference Gene Promoter Activation		References
	Log2-fold Change	*p*-adj	Log2-fold Change	*p*-adj	
**Differentially Expressed Risk Genes**					
ALAS1	-0.006	9.44E-01	0.174	2.15E-02	2
ANK2	0.143	2.42E-02	-0.097	1.78E-01	2
ASAH1	0.124	3.39E-02	0.073	2.80E-01	4
ASXL3	0.340	9.45E-03	0.165	2.87E-01	1
ATP13A2 (PARK9)	0.329	6.63E-05	0.312	3.67E-04	3
ATP1A3	0.427	8.45E-11	0.837	1.54E-39	3
ATP6V0A1	0.393	9.19E-09	0.222	2.99E-03	2
c5orf24	-0.044	5.77E-01	-0.176	1.70E-02	1
CAB39L	-0.659	7.87E-05	-0.427	1.86E-02	1
CAMK2D	-0.166	1.12E-02	-0.164	1.95E-02	1
CHD9	-0.295	1.36E-08	-0.167	3.25E-03	1
CHMP2B	-0.236	4.52E-03	-0.146	1.18E-01	2
CHRNB1	0.052	5.29E-01	0.228	2.58E-03	1
CRLS1	-0.176	1.71E-02	-0.038	6.99E-01	1
CTSB	0.112	3.15E-02	0.220	2.65E-05	4
CTSD	0.263	1.53E-05	0.201	2.05E-03	4
DCTN1	0.263	7.67E-06	0.060	4.18E-01	3
DDRGK1	-0.131	4.58E-02	-0.177	8.73E-03	2
DJ1 (PARK7)	0.175	1.01E-03	0.013	3.77E-01	3
DLG2	0.090	6.41E-01	-0.450	1.09E-02	1
DNAJC13	-0.264	9.99E-08	-0.158	3.20E-03	3
DYRK1A	-0.089	1.27E-01	0.330	1.71E-09	1
ELOVL7	-0.061	8.56E-01	1.309	2.02E-07	1
FAM171A1	0.402	2.61E-06	0.141	1.58E-01	2
FAM49B	0.122	4.81E-02	-0.210	8.87E-04	1
FAM47E-STBD1	-0.306	2.71E-04	-0.174	6.31E-02	1
FBRSL1	0.423	1.62E-08	0.230	5.06E-03	1
GAK (DNAJ26)	0.230	4.26E-03	0.366	7.68E-06	1
GBF1	0.121	5.98E-02	0.213	1.13E-03	1
GCH1	0.103	1.58E-01	-0.156	3.99E-02	1
GPNMB	-1.710	7.56E-12	0.106	7.52E-01	1
GRN	0.500	1.856E-13	0.286	8.96E-05	1
IP6K2	0.041	5.30E-01	-0.178	3.12E-03	1
ITPKB	-0.338	1.33E-06	0.297	5.31E-05	1
KAT8	0.179	8.76E-03	0.236	8.46E-04	4
KRTCAP2	0.007	9.39E-01	0.336	4.53E-06	1
LRRK2	-0.361	3.46E-02	-0.262	1.69E-01	1
LSM7	0.089	2.53E-01	0.229	2.68E-03	2
MAPT	0.738	4.56E-08	0.199	2.21E-01	2

MCCC1	-0.911	1.42E-02	0.218	3.24E-02	1
MED13	-0.117	4.52E-02	0.021	7.95E-01	1
MEF2D	-0.460	4.99E-01	0.198	5.01E-03	3
MEX3C	-0.116	4.18E-02	-0.115	5.69E-02	1
NDUFV1	0.140	2.78E-02	0.104	1.43E-01	3
NDUFV2	-0.051	4.60E-01	-0.191	3.32E-03	3
OGFOD2	-0.397	2.76E-05	0.086	4.68E-01	2
PAM	-0.782	1.97E-02	-0.023	7.75E-01	1
PINK1 (PARK6)	0.339	7.81E-06	0.197	1.91E-02	3
PMVK	0.202	1.83E-03	0.070	3.77E-01	1
RETREG3	0.412	2.38E-09	0.246	1.03E-03	1
RPS12	0.308	8.12E-09	0.169	3.84E-03	1
RPS6Kl1	0.276	1.39E-02	-0.0361	8.17E-01	1
SCAF11	-0.367	1.20E-07	-0.319	1.18E-05	1
SCARB2	-0.245	2.55E-06	-0.222	5.43E-05	1
SCN3A	-0.287	1.68E-03	-0.420	7.59E-06	2
SELENOT	-0.096	1.92E-01	-0.200	6.35E-03	3
SETD1A	0.283	7.76E-05	0.221	4.29E-03	1
SIPA1L2	-0.330	1.77E-07	0.085	2.63E-01	1
STK39	-0.206	1.03E-03	-0.173	1.03E-02	1
STMN2	0.323	2.52E-05	-0.258	1.74E-03	5
SYT4	-0.582	3.62E-02	-0.372	3.38E-11	2
TRPM7	-0.328	9.24E-07	-0.302	1.66E-05	3
UBTF	-0.121	1.53E-02	0.020	7.61E-01	1
UCHL1	0.128	1.73E-02	-0.151	7.53E-03	3
VAMP4	-0.195	2.54E-02	-0.264	3.52E-03	1
VPS13C	-0.501	5.93E-13	-0.404	2.41E-08	1
VPS35	-0.254	1.16E-02	-0.275	9.66E-03	4
ZNF184	-0.277	1.36E-03	-0.233	1.22E-02	2
ZNF646	0.158	3.21E-02	0.155	4.98E-02	2
**Risk Genes without Expression Change**					
BAG3	-0.211	7.47E-02	0.012	9.45E-01	1
BIN3	0.117	2.10E-01	0.134	1.82E-01	1
BST1	0.019	9.16E-01	-0.123	4.82E-01	1
CLCN3	-0.076	1.13E-01	-0.103	1.37E-01	1
COQ7	-0.071	4.47E-01	0.099	3.07E-01	2
DNAH17	-0.306	7.07E-02	0.510	4.41E-01	1
DRD4	0.125	7.98E-01	0.322	5.17E-01	3
FAM200B	-0.378	9.38E-02			2
FAM47E	-0.066	8.18E-01	-0.036	9.19E-01	1
FBXO7	-0.042	5.04E-01	-0.043	5.44E-01	4
FYN	0.174	1.41E-01	-0.046	7.27E-01	1
GALC	-0.113	1.19E-01	-0.089	2.69E-01	1
GBA	-0.057	4.69E-01	0.137	7.58E-01	2,3,4
GIGYF2 (PARK11)	-0.019	8.45E-01	-0.095	3.16E-01	3
INPP5F	-0.117	1.59E-01	-0.164	6.10E-02	1
KLHL7	-0.116	1.05E-01	-0.024	8.60E-01	2
KPNA1	-0.069	1.15E-01	-0.095	1.72E-01	1

LCORL	-0.467	6.24E-02	-0.076	4.08E-01	1
MAP4K4	0.078	1.45E-01	-0.039	5.47E-01	1
MBNL2	-0.361	7.87E-02	-0.094	3.04E-01	1
MECP2	0.074	3.43E-01	0.111	1.69E-01	3
MED12L	-0.219	1.97E-01	0.119	5.25E-01	1
MICU3	-0.494	9.93E-02	-0.038	8.31E-01	2
NCKIPSD	0.116	2.49E-01	0.177	8.57E-02	2
NOD2	-0.717	1.61E-01	-0.227	6.38E-01	1
NUCKS1	-0.075	2.33E-01	-0.071	3.08E-01	1
PACRG	0.013	9.64E-01	0.036	9.16E-01	3
PLA2G6	0.047	8.25E-01	-0.234	2.43E-01	3
PRKAG2	-0.104	3.53E-01	-0.207	6.32E-02	3
PRKN (PARK2)	0.315	4.64E-01	0.591	1.82E-01	3
RAB29	-0.313	8.08E-02	-0.115	1.67E-01	1
RIMS1	-0.840	5.15E-02	-0.112	6.83E-01	1
RNF141	-0.506	5.66E-02	-0.056	5.88E-01	1
SATB1	0.166	9.99E-02	-0.136	7.18E-02	1
SLC17A5	0.144	1.22E-01	-0.173	8.36E-02	4
SMPD1	0.029	8.09E-01	0.154	1.67E-01	4
SNCA	0.134	9.86E-02	0.070	4.68E-01	1
SORBS3	0.436	6.06E-02	-0.023	8.12E-01	2
SPPL2B	0.053	1.98E-01	0.055	6.80E-01	1
STX1B	0.072	7.20E-01	-0.236	2.29E-01	3
SYT17	0.299	1.84E-01	0.162	4.14E-01	1
TAF1	-0.098	2.05E-01	-0.106	1.93E-01	3
TMEM175	0.104	2.24E-01	-0.090	5.98E-01	1
TMEM230	-0.101	8.96E-02	-0.035	6.38E-01	3
UBAP2	0.439	5.74E-02	0.063	4.38E-01	1
WNT3	-0.099	1.78E-01	-0.150	2.08E-01	1
**Risk Genes not Expressed in SHSY5Y Cells**					
ADRB2					3
CASC16					1
CD19					1
CRHR1					1
FCGR2A					1
FGF20					1
GABP					1
HLADQA1					4
HLA-DRB5					1
HLADRB6					2,4
HP					3
IL1R2					2
ITGA8					1
KCNIP3					1
KCNS3					1
MIPOL1					1
NR4A2					3
RAB7L1					4

RIT2	1
SH3GL2	1
SNCAIP	3
SPTCCB	1
TMEM163	1
TMEM229B	2
TOX3	2
TRIM40	1

p, adjusted by Benjamini-Hochberg procedure; upregulation or repression relative to transfection with scrambled sgRNA are highlighted in green or red, respectively.

Reference List

1. Nalls MA, Blauwendraat C, Vallerga CL et al. Identification of novel risk loci, causal insights, and heritable risk for Parkinson's disease: a meta-analysis of genome-wide association studies. Lancet Neurol 2019;18(12):1091-1102.

2. Chang D, Nalls MA, Hallgrimsdottir IB et al. A meta-analysis of genome-wide association studies identifies 17 new Parkinson's disease risk loci. Nat Genet 2017;49(10):1511-1516 3.

3. https://ghr.nlm.nih.gov/condition/parkinson-disease#resources

4. Billingsley KJ, Bandres-Ciga S, Saez-Atienzar S, Singleton AB. Genetic risk factors in Parkinson's disease. Cell Tissue Res 2018;373(1):9-20.
5. Melamed Z, Lopez-Erauskin J, Baughn MW et al. Premature polyadenylation-mediated loss of stathmin-2 is a hallmark of TDP-43-dependent neurodegeneration. Nat Neurosci 2019;22(2):180-190.

Table S8   Primers for RT-PCR studies

Primers for the detection of dCas9

dCAS9 forward	5´AGGTGGCGTACCATGAAAAGTA 3´
dCAS9 reverse	5´GCTGTTGTCTGGGTTCAGGT 3´

RT/PCR amplification primers for *PPARGC1A* transcripts

PPARGC1A Exon B1 forward	5´TACAACTACGGCTCCTCCTGG 3´
PPARGC1A Exon B4 reverse	5´TACCCTTCATCCATGGGGCTC 3´
PPARGC1A Exon B5 forward	5´CCTGGCTGCTGCTTTGGTA 3´
PPARGC1A Exon 1 forward	5´CGTGGGACATGTGCAACCAGG 3´
PPARGC1A Exon 2 reverse	5´GCTGTCTGTATCCAAGTCGT 3´
PPARGC1A Exon 5 forward	5´TCACACCAAACCCACAGAGA 3´
PPARGC1A Exon 6A reverse	5´GGTCACTGGAAGATATGGCACATTA 3´
PPARGC1A Exon 2 forward	5´GTACAACAATGAGCCTTCAAAC 3´
PPARGC1A Exon 3ext reverse	5´TACCATCATACCCATGCAGCG 3´
PPARGC1A Exon 12 forward	5´AGCTGTACTTTTGTGGACGCAAG 3´
PPARGC1A Exon 13 reverse	5´GGTGGAAGCAGGGTCAAAGTC 3´
RPLP0 forward	5´GGCACCATTGAAATCCTGAGTGAT 3´
RPLP0 reverse	5´TTGCGGACACCCTCCAGGAAGC 3´

Primers for *PPARGC1A* target transcripts

NEUROG2 forward	5´GGAGTACCCTGATGAGATCGAG 3´
NEUROG2 reverse	5´GAGCAGCACTAACACGTCCTC 3´
CHGA forward	5´GTTGAGGTCATCTCCGACACAC 3´
CHGA reverse	5´ACCGCTGTGTTTCTTCTGCT 3´
IGF2 forward	5´ACACCCTCCAGTTCGTCTGT 3´
IGF2 reverse	5´GGGGTATCTGGGGAAGTTGTC 3´

ASIC1 forward	5 ´GAACCTCCTTTCATCGACCAGC 3 ´
ASIC1 reverse	5 ´GCAGTTCTCCACCAGGTAGC 3 ´
EPAS1 forward	5 ´GGGCAGATCCAATACCCAGTG 3 ´
EPAS1 reverse	5 ´CAGCTTGTTGGAGAGGGCTAC 3 ´
MELTF forward	5 ´ACCACTGCGTCCAGCTCAT 3 ´
MELTF reverse	5 ´GCCTTTCAGGGTGTCAATGGTC 3 ´
VGF forward	5 ´GACCCTCCTCTCCACCTCTC 3 ´
VGF reverse	5 ´GAGCTGAGAGGAGGAGGCTG 3 ´
TNFRSF12A forward	5 ´CCGCAGGAGAGAGAAGTTCAC 3 ´
TNFRSF12A reverse	5 ´CTTGTGGTTGGAGGAGCTTGG 3 ´
IGFBP3 forward	5 ´GTCCCTGCCGTAGAGAAATGG 3 ´
IGFBP3 reverse	5 ´GCTGCCCATACTTATCCACACA 3 ´
RPLP0 forward	5 ´GGCACCATTGAAATCCTGAGTGAT 3 ´
RPLP0 reverse	5 ´TTGCGGACACCCTCCAGGAAGC 3 ´

Supplementary Figures

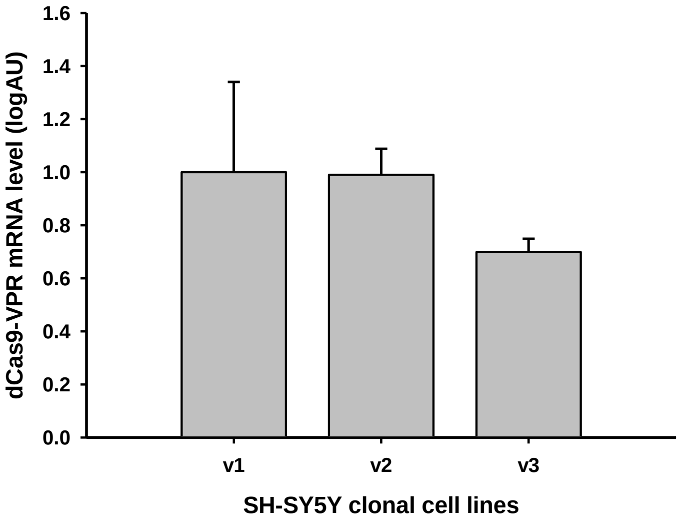

Figure S1. mRNA expression levels of dCas9-VPR in clonal SH-SY5Y cell lines v1-v3 expressed in logAU, not significantly different by ANOVA.

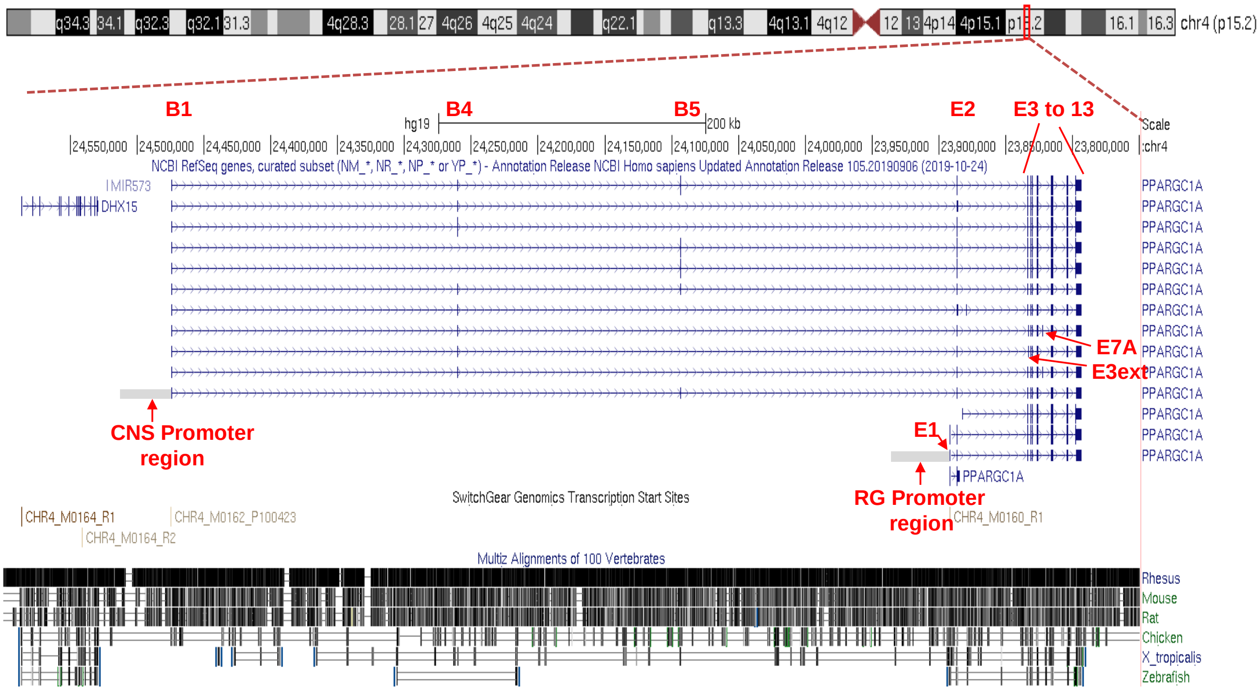

Figure S2. Genomic *PPARGC1A* locus (depicted in reverse orientation to its natural location at Chromosome 4p15.2) showing CNS-specific exons B1, B4 and B5 and CNS and reference gene (RG) promoters in the context of their predicted (SwitchGear) transcription start sites. The RG promoter region as well as CNS-specific exons are conserved in rodents, but transcripts containing exon B5 are not detected in rodents.

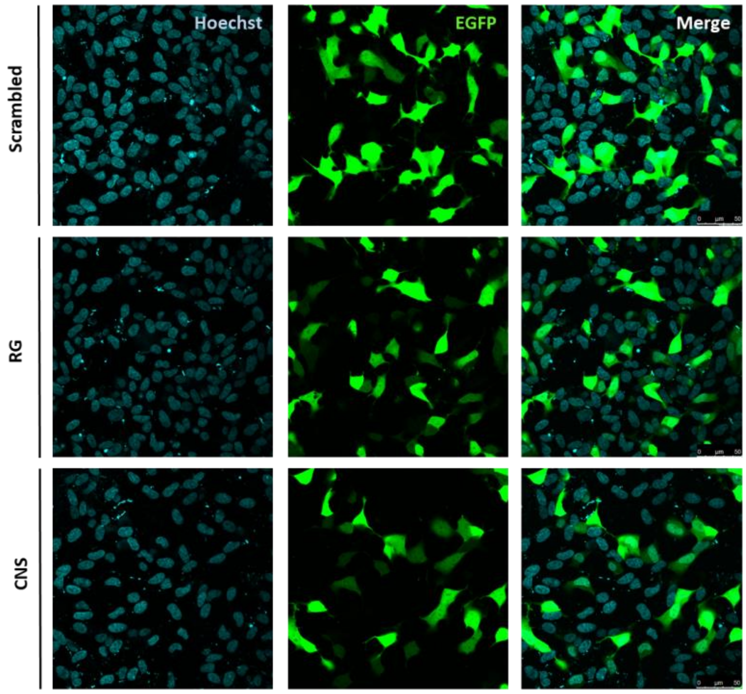

Figure S3. Fluorescent imaging of SH-SY5Y dCAS9-VPR positive clonal cells transfected with plasmids co-expressing scrambled, Reference gene (RG) promoter and CNS promoter sgRNAs and EGFP. From left to right: fluorescent signals of nuclei counterstained with Hoechst (cyan), EGFP and merged image.

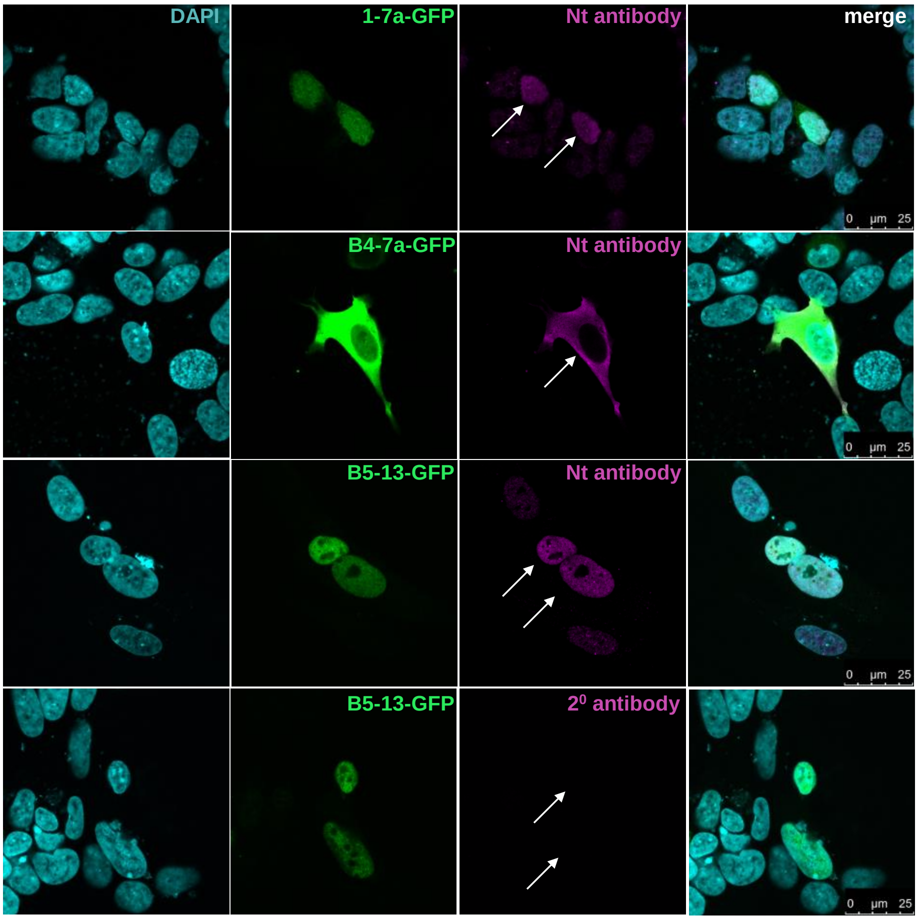

Figure S4. Specificity of the antibody raised against the N-terminal (Nt) region of reference PGC1-α. From left to right: Fluorescent signals of cellular nuclei counterstained with 4',6-diamidino-2-phenylindole (DAPI, cyan), EGFP (green) fused to the C-terminus of the indicated PGC-α variants, Cy5 (NT antibody, magenta) and corresponding merged image. The Nt antibody detects E1-7a-EGFP as well as B4-7a-EGFP and B5-13-EGFP in transfected SH-SY5Y cells. In adjacent non-transfected cells or in cells incubated only with the secondary antibody, the Cy5 signal was minimal or absent. The arrows indicate transfected cells.

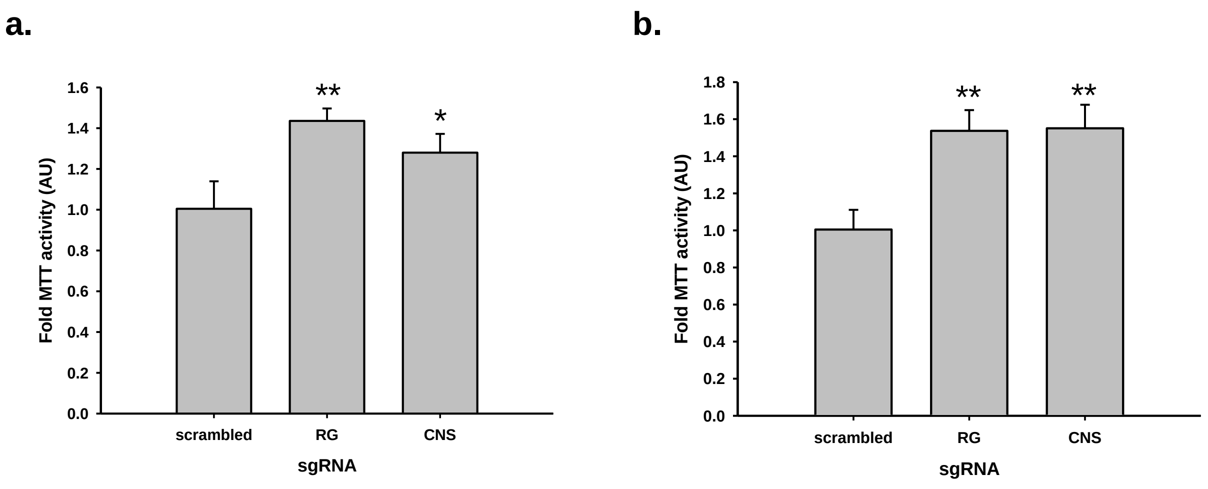

Figure S5. Metabolic activity of clonal dCAS9-VPR expressing SH-SY5Y cells cultured for 20 (**a**) or 42 h (**b**)as ascertained by the MTT assay; **P*<0.01; ***P*<0.001.

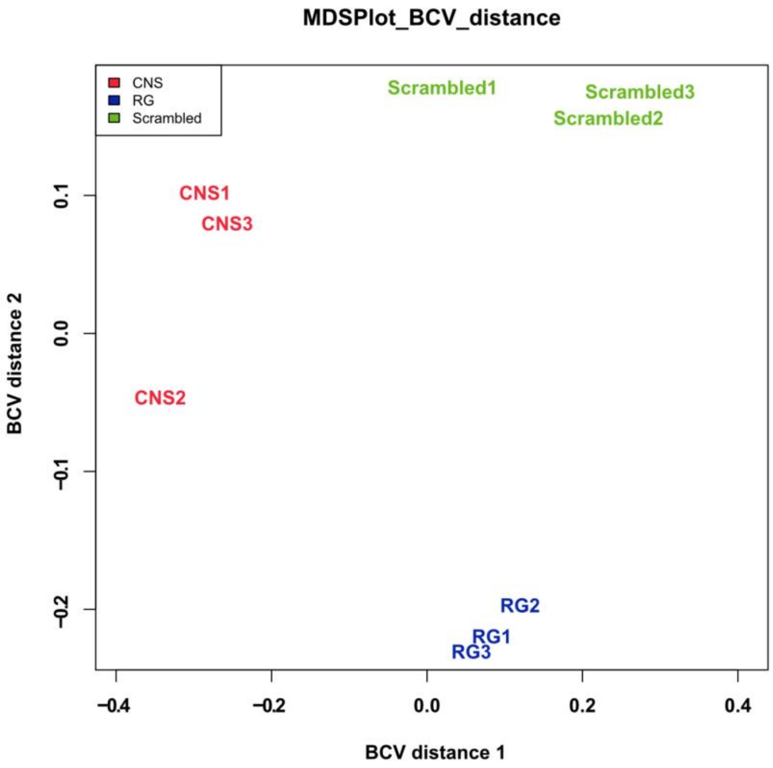

Figure S6. Multidimensional Scaling (MDS) plot showing clustering of the respective biological replicates.

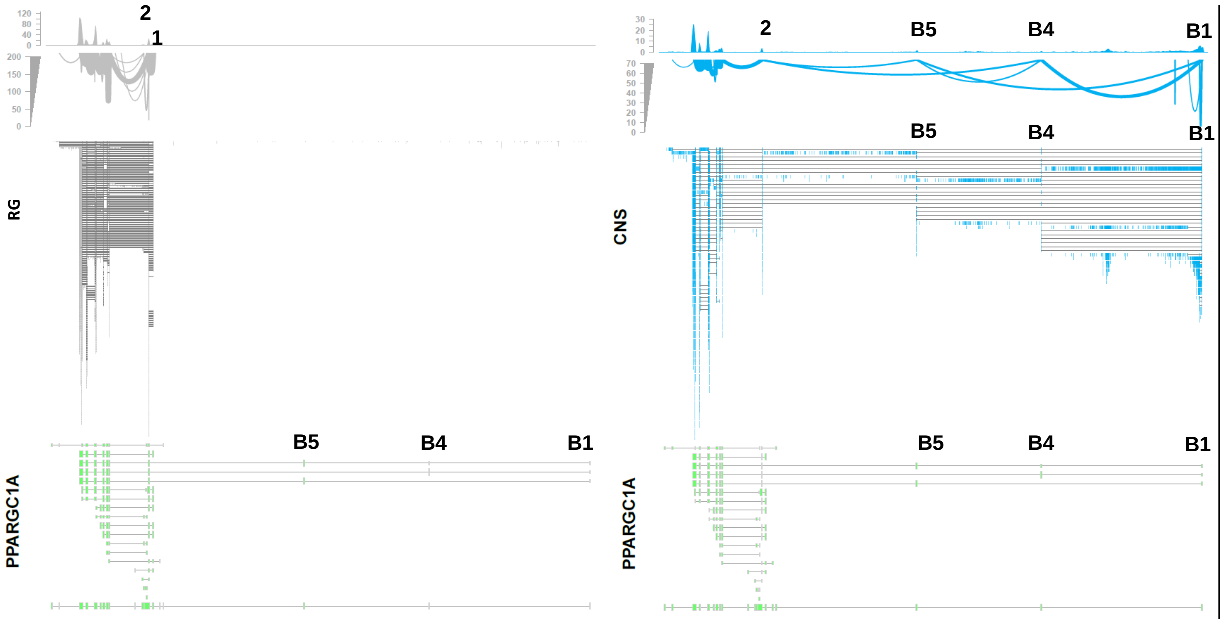

Figure S7. Sashimi plots of RG (left) and CNS (right); *PPARGC1A* transcripts generated by activation of the RG or CNS promoters as described in Fig. 3. Reads are shown in the middle panels and transcript structures are aligned at the bottom.

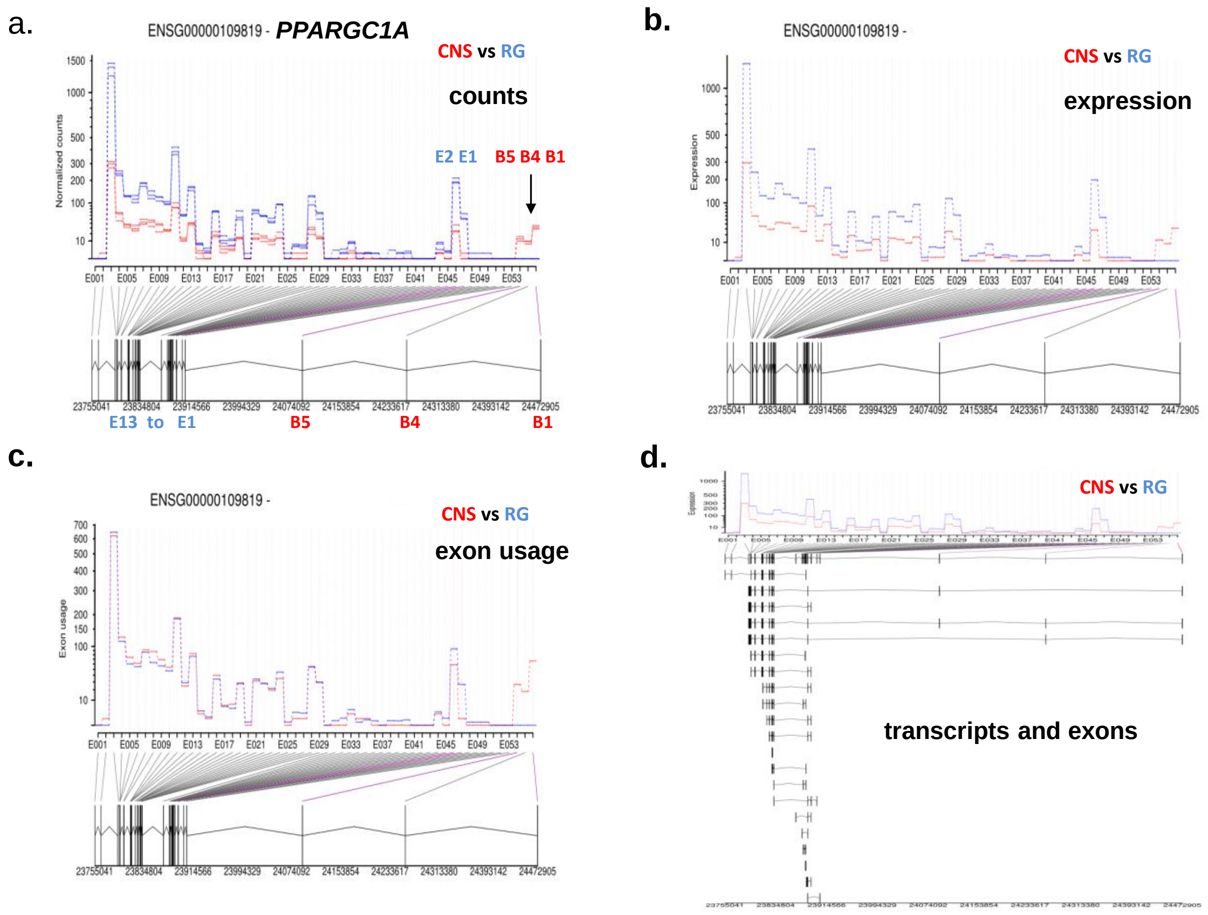

Figure S8. Expression and exon usage of RG and CNS exon bins in cells transfected with sgRNAs targeting the respective promoter. (**a**) Comparison of replicate counts between transcripts generated by activation of CNS and RG promoters. (**b**) Comparison of the expression of exon bins. (**c**) Comparison of exon usage. (**d**) Comparison of aligned exons and visualized transcript structures. Arrows indicate the CNS specific exons.

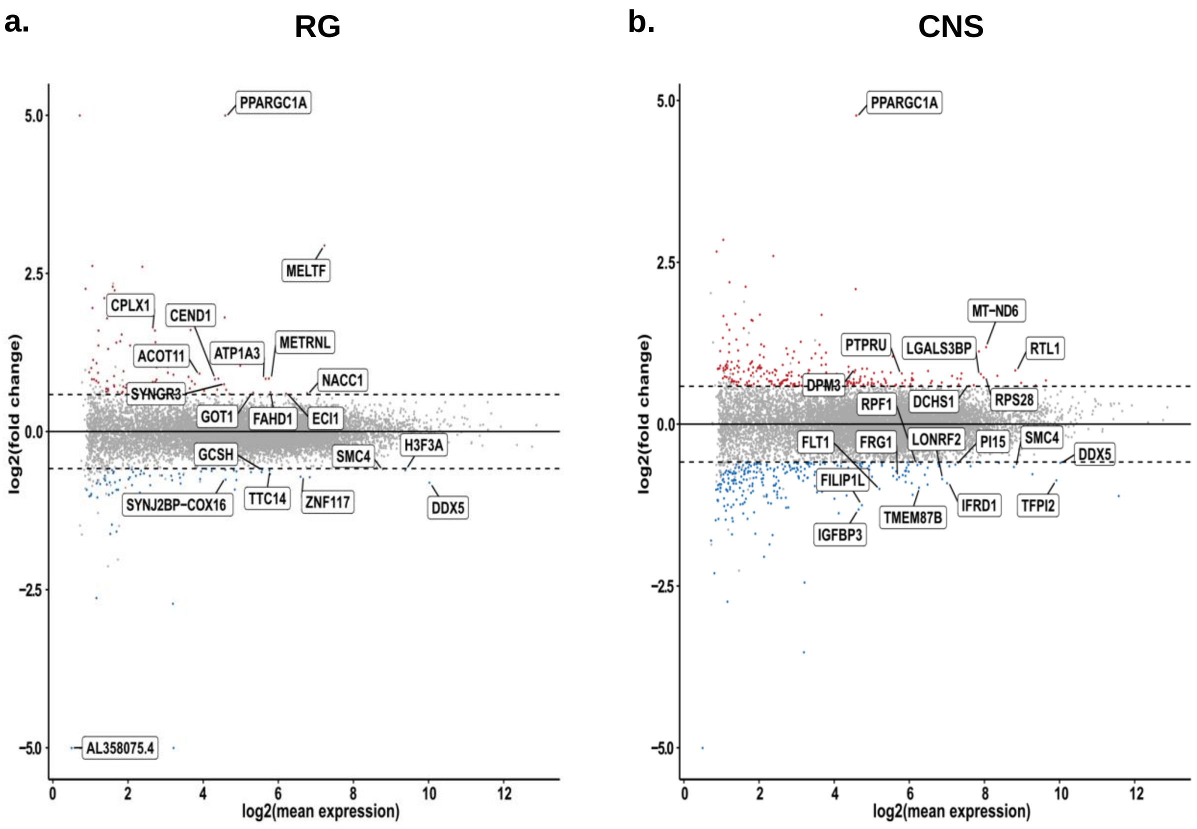

Figure S9. (**a**) and (**b**) MA plots of differentially expressed genes (DEGs) in cells with RG or CNS activated promoters, respectively, in comparison to cells transfected with scrambled sgRNAs. Top 20 most DEGs are highlighted.

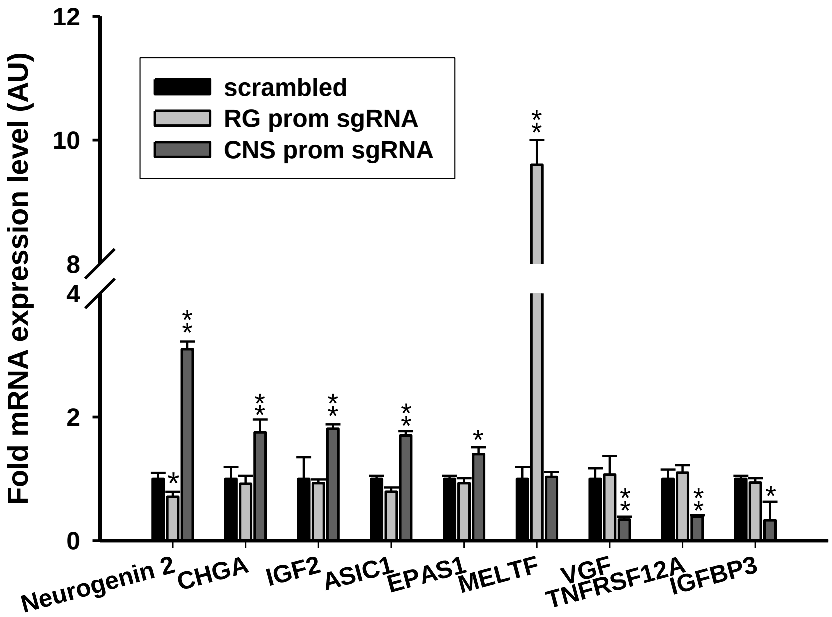

Figure S10. Verification of selected DEGs in clonal dCAS9-VPR - expressing SH-SY5Y cells after activation of RG or CNS promoters or transfection of scrambled sgRNAs (control) by qRT-PCR; ANOVA, Tukey‘s post hoc comparison *P<0.01, **P<0.001 vs. control.

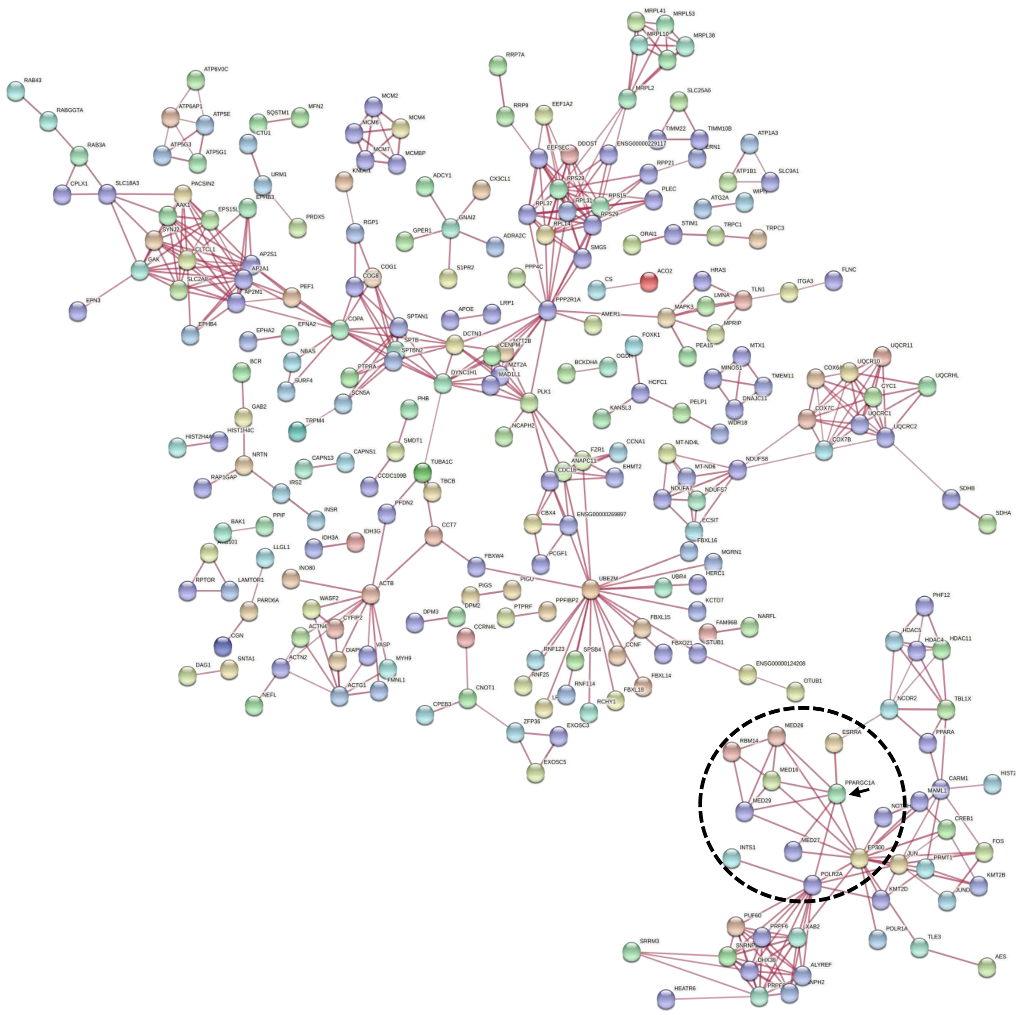

Figure S11. Predicted physical interactions of proteins translated from all genes upregulated after activation of the RG promoter. Nodes represent proteins, edges show high confidence interactions (interaction score >0.7). Disconnected proteins are not shown. The circle indicates the network that includes PPARGC1A shown by the arrow.

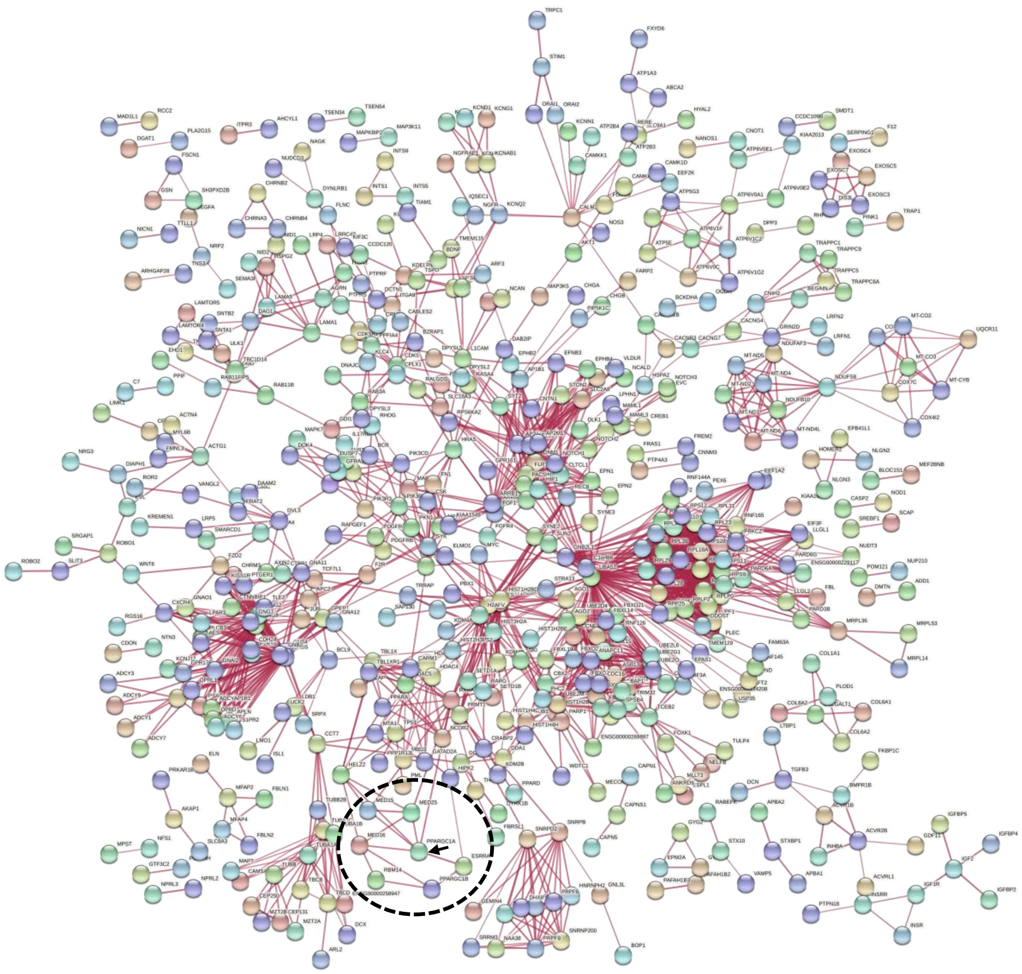

Figure S12. Predicted physical interactions of proteins translated from all genes upregulated after activation of the CNS promoter. Nodes represent proteins, edges show high confidence interactions (interaction score >0.7). Disconnected proteins are not shown. The circle indicates the network that includes PPARGC1A shown by the arrow. The software does not recognize PPARGC1A isoforms.

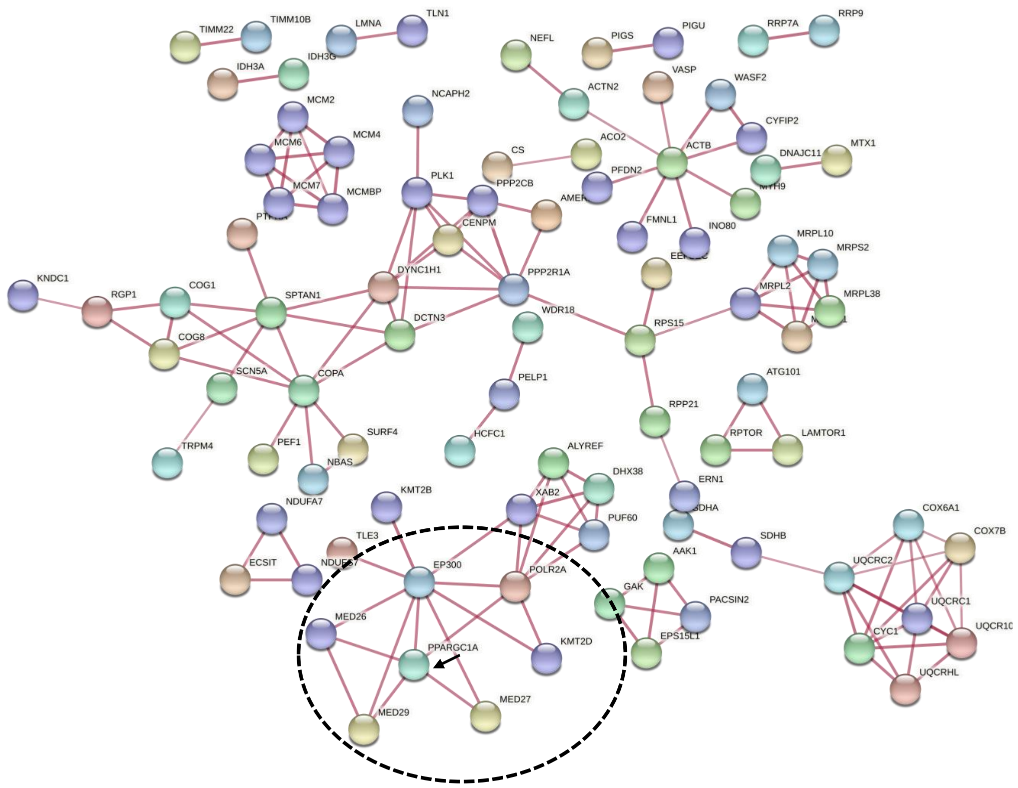

Figure S13. Predicted physical interactions of proteins translated from all genes selectively upregulated after activation of the RG promoter (see Fig. 4a). Nodes represent proteins, edges show high confidence interactions (interaction score >0.7). Disconnected proteins are not shown. The circle indicates the network that includes PPARGC1A shown by the arrow.

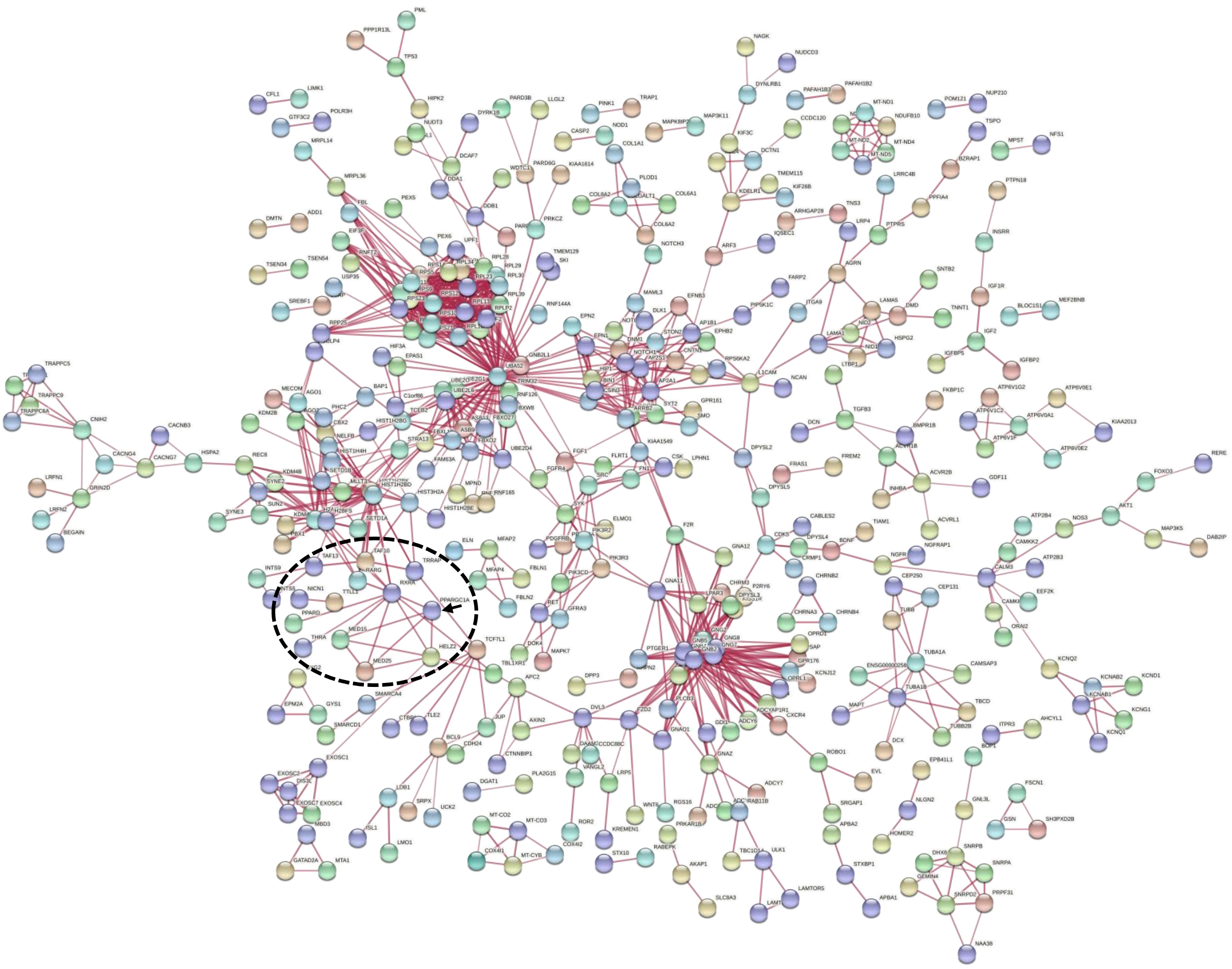

Figure S14. Predicted physical interactions of proteins translated from all genes selectively upregulated after activation of the CNS promoter (see Fig. 4a). Nodes represent proteins, edges show high confidence interactions (interaction score >0.7). Disconnected proteins are not shown. The circle indicates the network that includes PPARGC1A shown by the arrow. The software does not recognize PPARGC1A isoforms.

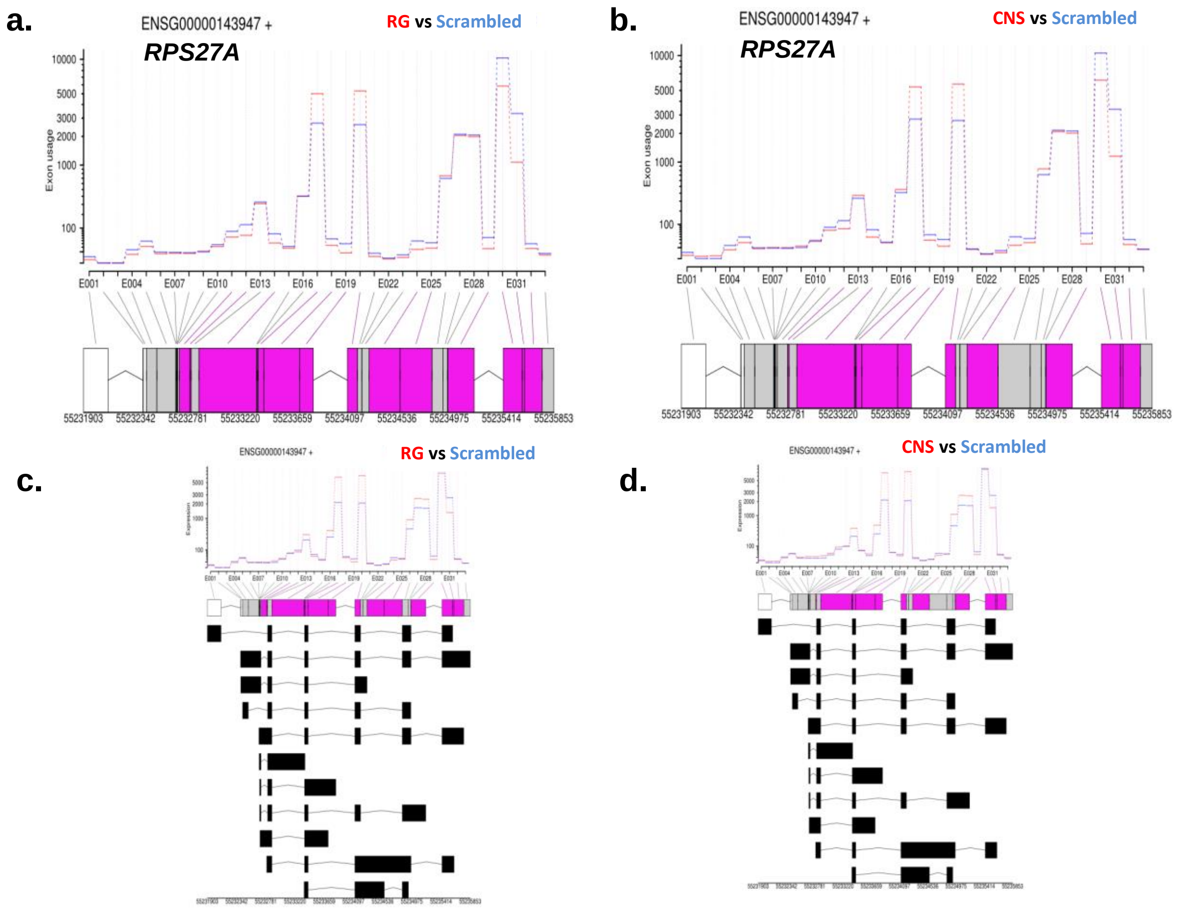

Figure S15. Exon usage of *RPS27A* without significant differences between RG and CNS promoter activation, but distinct from transfections of scrambled sgRNA (control). (**a**) and (**b**) Comparisons of exon usage between activation of RG or CNS promoters and control. (**c**) and (**d**) Comparisons of exon bin expression between activation of RG or CNS promoters and control, respectively; potential transcripts are shown below in relation to exon bins; differential expression and/or exon usage between pairwise comparisons is indicated by the purple boxes representing the exon bins, shown on the abscissa.

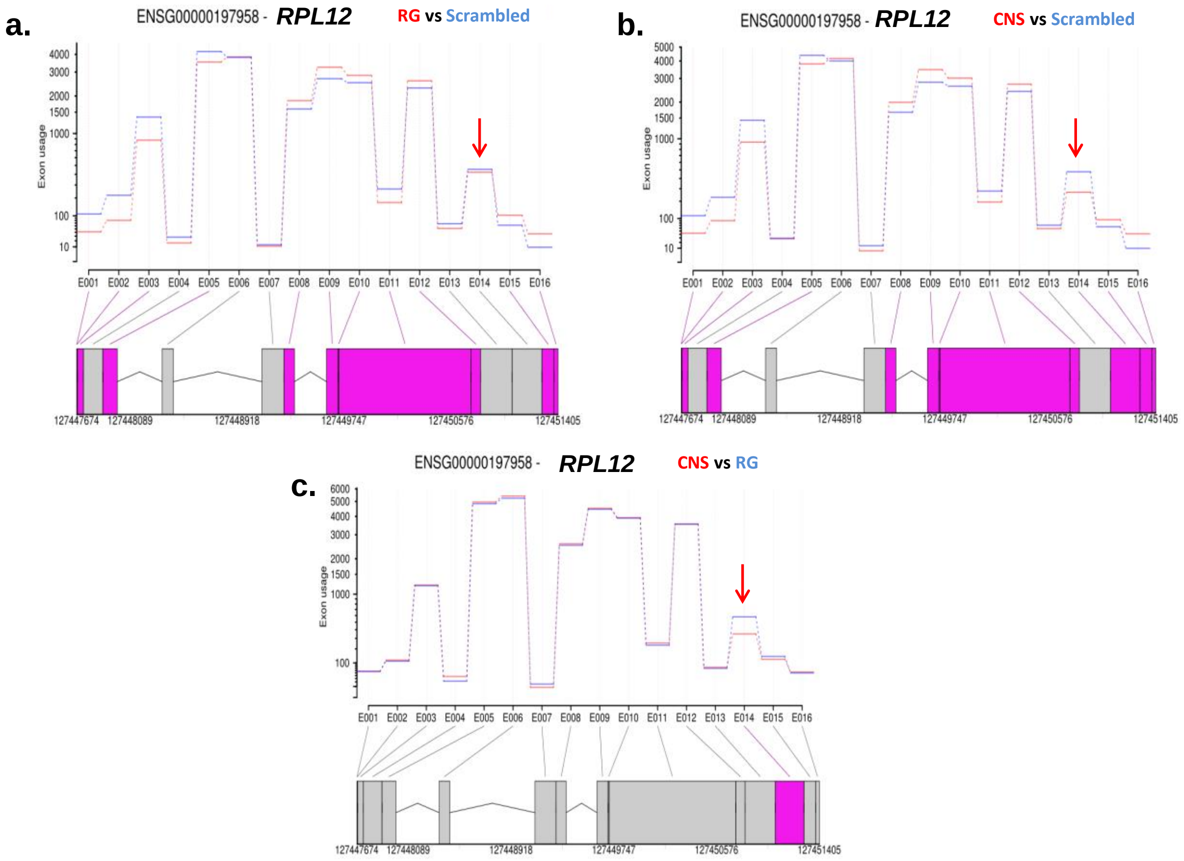

Figure S16. Exon usage of RPL12 (**a**) and (**b**). Activation of RG and CNS promoters, respectively, reveals comparable changes in *RPL12* exon usage to transfections of scrambled sgRNAs (control) with the exception E014. (**c**) The difference in exon usage is clearly visible (see arrows) in the comparison of CNS and RG promoter activation, different exon usage between pairwise comparisons is indicated by purple boxes representing the affected exon bins.

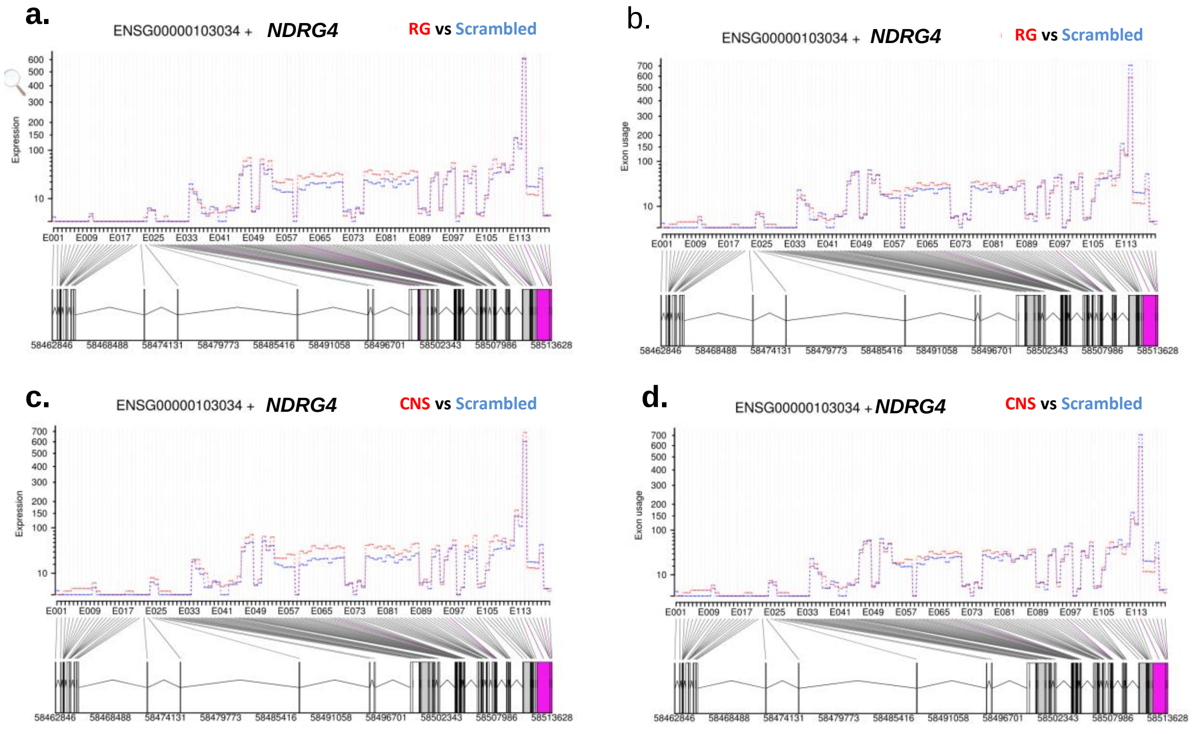

Figure S17. Exon usage of *NDRG4;* exon usage is similar for RG and CNS promoter activation but is distinct from transfection of scrambled sgRNA (control). (**a**) and (**b**) Comparisons of expression and exon usage between activation of RG promoter and control. (**c**) and (**d**) Comparisons of expression and exon usage between activation of CNS promoter and control; different expression and/or exon usage between pairwise comparisons is indicated by the purple boxes representing the exon bins, shown on the abscissa.

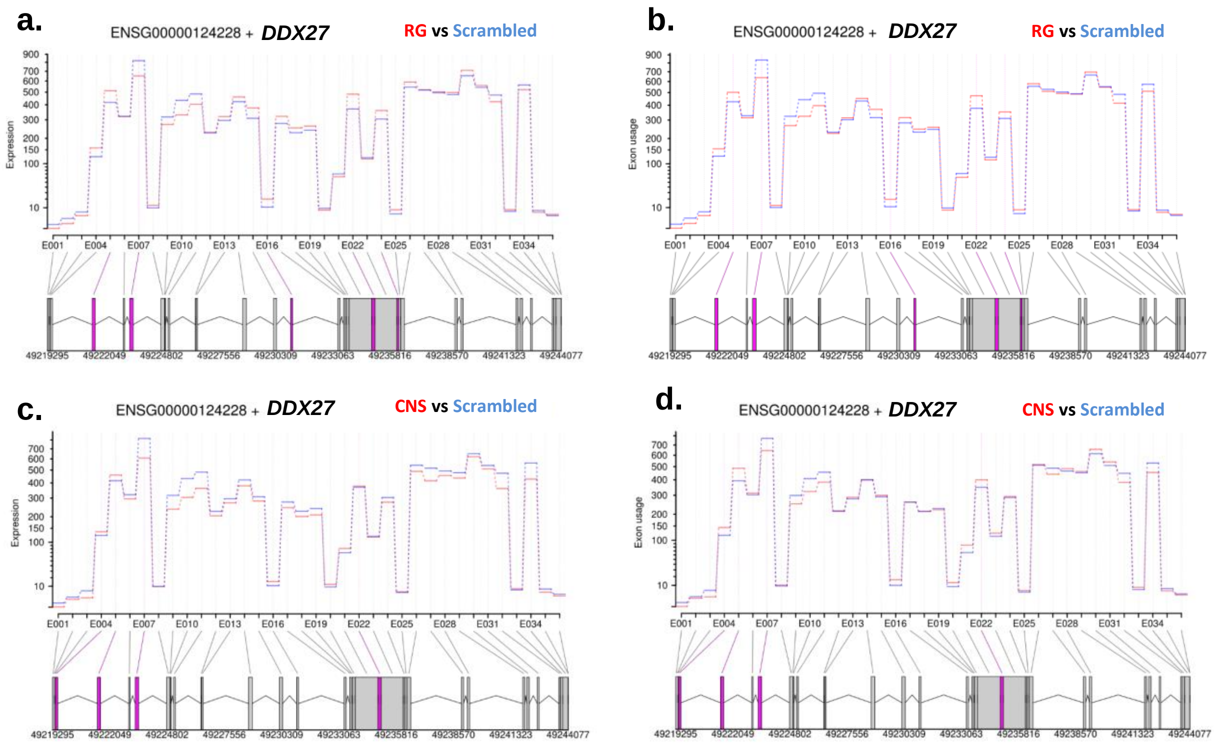

Figure S18. Exon usage of *DDX27; e*xon usage is similar for RG and CNS promoter activation, but is distinct from transfection of scrambled sgRNA (control). (**a**) and (**b**) Comparison of expression and exon usage between activation of RG promoter and control, respectively. (**c**) and (**d**) Comparisons of expression and exon usage, respectively, between activation of CNS promoter and control; different expression and/or exon usage between pairwise comparisons is indicated by the purple boxes representing the exon bins, shown on the abscissa.
